# Ecological Analysis of Spatiotemporal Patterns in Brain Tumor Incidence and Cellular Infrastructure Growth in the United States

**DOI:** 10.3390/cancers18213419

**Published:** 2026-10-22

**Authors:** János A. Barbero, Prashin Unadkat, Daniel G. Eichberg, Randy S. D’Amico, Daniel M. Sciubba, Michael Schulder, Jung Park

**Affiliations:** 1 Donald and Barbara Zucker School of Medicine at Hofstra/Northwell, Hempstead, NY 11549, USA; 2 The Feinstein Institutes for Medical Research, Manhasset, NY 11030, USA; 3 Department of Neurosurgery, Baylor College of Medicine, Houston, TX 77030, USA; unadkatprashin@gmail.com; 4 Northwell, New Hyde Park, NY 11042, USA; deichberg@northwell.edu (D.G.E.); rdamico8@northwell.edu (R.S.D.); dsciubba1@northwell.edu (D.M.S.); mschulder@northwell.edu (M.S.); jpark27@northwell.edu (J.P.); 5 Department of Neurosurgery, North Shore University Hospital, Manhasset, NY 11030, USA

**Keywords:** radiofrequency electromagnetic fields, glioblastoma, cellular infrastructure, ecological study, SEER, brain tumors, cell towers, RF-EMF, public health, cancer incidence

## Abstract

**Background/Objectives:** The widespread growth of wireless technologies has made ambient exposure to radiofrequency electromagnetic fields (RF-EMF) ubiquitous. Previous studies have examined associations between mobile phone use and primary malignant brain tumor risk, yet the oncologic impact of ambient exposure remains unclear. **Methods:** We linked cellular infrastructure density from the OpenCelliD geodatabase to county-level brain tumor incidence in the National Cancer Institute’s Surveillance, Epidemiology, and End Results registry across 884 counties covering 46% of the U.S. population. Deployment followed a sigmoid growth pattern with an inflection in 2016; we compared infrastructure density and incidence ratios from 2011–2015 to 2016–2020, adjusting for baseline rates. Legacy cell tower infrastructure and LTE were examined separately, with vestibular schwannoma as a negative control, confounder adjustment, repeated-measures panels, and age stratification. **Results:** Growth in infrastructure density correlated with growth in total primary malignant brain tumors (r = 0.19, *p* = 2.2 × 10^−8^, 95% CI 0.122 to 0.250) and glioblastoma (r = 0.14, *p* = 5.5 × 10^−5^, 95% CI 0.070 to 0.200). The total-tumor association persisted after regressing out glioblastoma, though the non-GBM component attenuated to non-significance after full confounder adjustment. Vestibular schwannoma showed no significant association. The GBM association was significant for legacy cell tower infrastructure (r = 0.104, *p* = 0.0024, 95% CI 0.037 to 0.170) but non-significant for LTE, persisted after confounder adjustment, and was significant only in adults aged 70 and older. The GSM definition was post hoc, and its association was model-dependent. In the first-difference (repeated-measures) analysis, GBM was not significantly associated with legacy cell tower infrastructure (coefficient = 0.014, *p* = 0.70, 95% CI −0.060 to 0.088), while the association with all towers did not reach statistical significance (coefficient = 0.13, *p* = 0.052, 95% CI −0.001 to 0.251). **Conclusions:** County-level growth of cellular infrastructure is modestly associated with primary malignant brain tumor incidence. The legacy association was significant only in adults aged 70 and older; LTE and vestibular schwannoma showed no significant association. These findings are hypothesis-generating; causation is not established.

## Simple Summary

Brain cancer rates have increased in some parts of the world, raising questions about whether the growing density of cell phone towers might play a role. Studies of individual phone use can be affected by errors in people’s recollections. This study instead examined whether U.S. counties with faster growth in tower density also experienced greater increases in brain cancer. We analyzed information from 884 counties representing almost half of the U.S. population, combining tower installation records with brain tumor diagnoses from the National Cancer Institute. Counties where older-generation (2G) tower density grew more quickly had modestly greater increases in glioblastoma, the most common malignant brain tumor. This association remained after accounting for differences in socioeconomic conditions, healthcare, and environmental factors. Newer-generation (4G) towers showed no significant association with glioblastoma, and overall tower growth showed no significant association with vestibular schwannoma. These findings do not establish cause and effect, but suggest that radiofrequency exposure in the surrounding environment from cell tower infrastructure deserves further study.

## 1. Introduction

The exponential increase in human exposure to radiofrequency electromagnetic fields (RF-EMF) stems primarily from the widespread adoption of wireless communication technologies in recent decades. Mobile phones are now owned by approximately 80% of the global population [1]. This increasing exposure has raised concerns about potential health implications, particularly regarding primary malignant brain tumor development [2,3].

The World Health Organization’s International Agency for Research on Cancer (IARC) classifies RF-EMF as ‘possibly carcinogenic to humans’ (Group 2B), leading regulatory bodies to establish varying safety thresholds and exposure guidelines [4,5]. This classification was largely based on the findings from two landmark human studies. The INTERPHONE study [6] reported no overall risk of glioma from routine cell phone use but found an elevated risk among the heaviest users (>1640 h cumulative call time), particularly on the side of regular phone use and within the temporal lobes where RF-EMF exposure is highest, although the study authors caution that selection and recall biases could explain this finding. Similarly, the Swedish pooled analysis [7] demonstrated increased risks with long-term use (>10 years) and ipsilateral exposure. Some studies have found physiological correlates of RF-EMF with brain cellular metabolism, thus supporting a possible oncogenic mechanism [8,9,10]. Overall, the evidence appears to suggest no increased risk for primary malignant brain tumor development in the great majority of mobile phone users. The studies that have addressed this question are largely complicated by difficulties in ascertaining the extent of mobile phone use with the phone held close to the ear. Furthermore, any such increased risk is more likely to have been associated with earlier generations of cellular technology that yielded much higher RF exposures to phone users.

Cell towers can also be a significant source of RF-EMF, especially to those residing in close proximity [11]. To provide geographically equitable coverage and support higher spectrum connectivity, the Federal Communications Commission (FCC) has policies facilitating cellular tower deployment, particularly in non-urban areas [12,13,14]. However, the potential epidemiological implications of this infrastructure expansion remain unclear.

Several geographic and ecological analyses have addressed this question at the population level. Time-trend analyses in Nordic countries found no overall increase in brain tumor incidence despite rising mobile phone use [15], and Australian analyses spanning nearly three decades found no increase in brain tumor incidence [16]. Increases in glioblastoma incidence have nonetheless been documented in both UK [17] and US registries [18]. This divergence from the trend of stable overall brain tumor rates remains incompletely explained. Interpreting any infrastructure–incidence association in this literature is complicated by the fact that urbanization drives both cellular deployment and diagnostic ascertainment. Prior aggregate analyses have used national-level subscription or adoption metrics that do not resolve geographic variation in ambient exposure or distinguish among cellular technology generations, while individual-level studies largely relied on self-reported phone use, which is subject to recall bias and systematic measurement error in both duration and laterality of exposure.

To address these limitations we conducted a population-based ecological study to investigate the temporal changes in primary malignant brain tumor incidence and their association with changes in cell tower deployment patterns across 884 United States counties. We examined whether any observed associations persisted after adjustment for multiple confounders, were robust to repeated-measures analyses and time lag sensitivity analyses, varied by technology generation, and differed by age group.

## 2. Materials and Methods

### 2.1. Study Design

**Figure 1:**
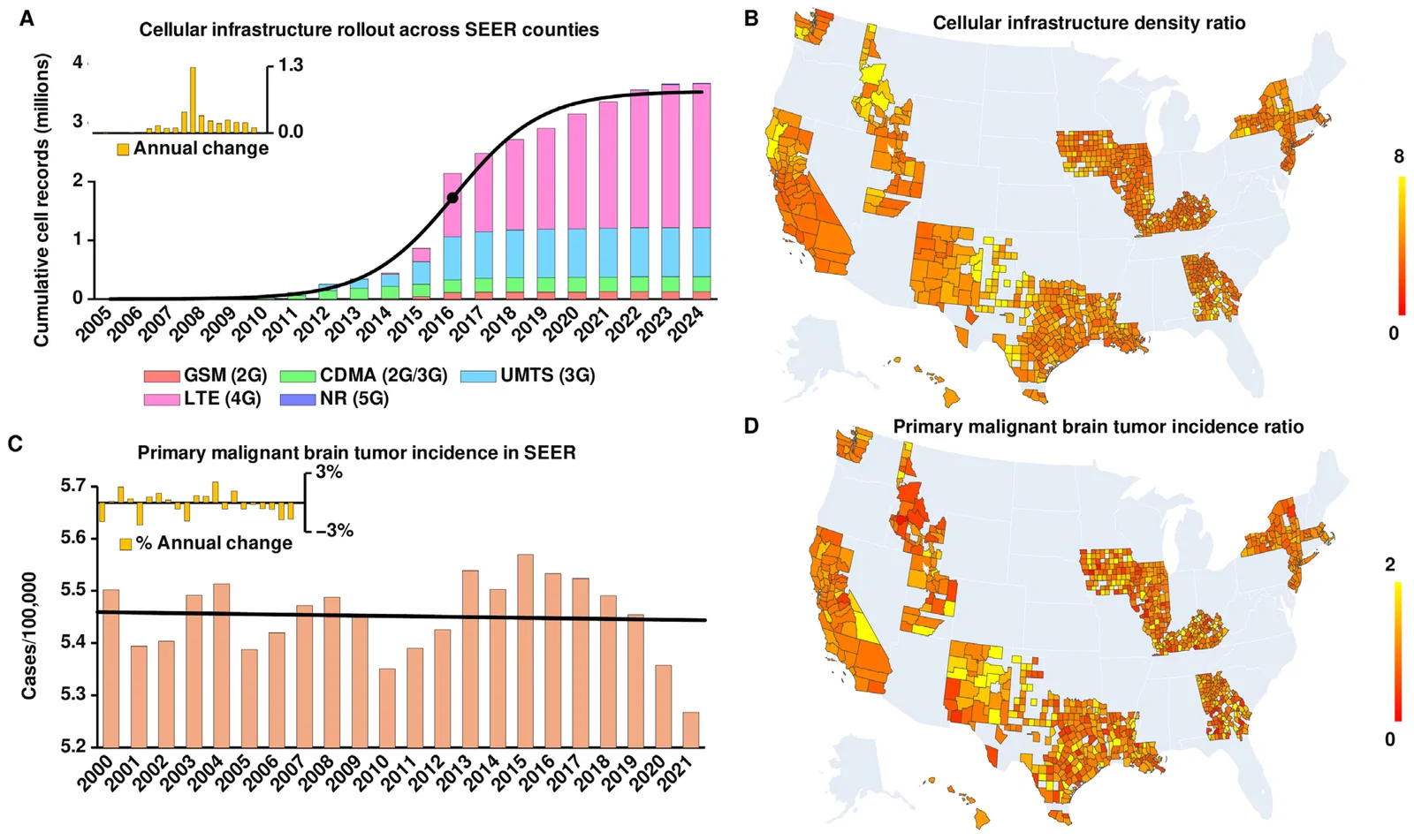
Cellular infrastructure deployment and primary malignant brain tumor incidence. (**A**) Cumulative cell records from OpenCellID (2005–2024) across counties included in the National Cancer Institute’s Surveillance, Epidemiology, and End Results (SEER) program’s county-level data, stratified by technology generation. The cumulative number of records follows a sigmoid distribution (black curve) with an inflection point (2016, black dot) that defines pre- and post-2016 study epochs. (**B**) Geographic variation in cellular infrastructure growth across SEER counties, shown as the ratio of cumulative towers/km^2^ between post-inflection (2016–2020) and pre-inflection (2011–2015) epochs. Counties with ratios greater than 8 are colored yellow. (**C**) Annual total primary malignant brain tumor incidence per 100,000 people across counties in the most recent SEER county-level data available (2000–2021) shows a steady rate (black trendline). (**D**) Corresponding geographic variation in primary malignant brain tumor incidence growth, shown as the ratio of average cases per 100,000 people between epochs. Counties with ratios greater than 2 are colored yellow.

We conducted a population-based ecological study examining the relationship between changes in cellular infrastructure density and changes in brain tumor incidence. Incidence of primary malignant brain tumors, glioblastomas (GBM), and vestibular schwannomas (VS) was drawn from the National Cancer Institute’s Surveillance, Epidemiology, and End Results (SEER) database [19], while cellular infrastructure location data was obtained from OpenCelliD [20]. To identify meaningful study periods, we analyzed cellular infrastructure deployment patterns across the counties in SEER by fitting a sigmoid logistic function to cumulative tower counts (Figure 1A). The sigmoid function’s midpoint (2016) indicates the inflection point for cellular infrastructure deployment. Based on this, we chose pre-inflection (2011–2015) and post-inflection (2016–2020) periods of equal length for further analysis. Population-normalized primary malignant brain tumor incidence remained flat throughout the SEER registry period (Figure 1C), while GBM incidence showed a steady linear increase. Full methodological details are given in the Supplementary Methods.

Population data from the U.S. Census Bureau [21,22] aligned with SEER except for some exclusions, leaving 1069 counties across 15 states for analysis (Figure 1B). Connecticut was excluded due to its 2019 county reorganization, Alaska’s registry is not county-coded, and Washington data cover Seattle–Puget Sound.

We calculated cumulative cellular infrastructure density (towers/km^2^) and average annual crude (not age-adjusted) tumor incidence rates (cases/100,000 people) per county, then computed their ratios between study periods. Of 1069 counties, 179 were excluded for zero GBM cases in one or both epochs; none were excluded for zero towers or population, leaving 890 counties before outlier exclusion (Figure 1D). The excluded counties were small rural counties (median 2020 population 6512), accounting for 0.9% of the 2020 population.

### 2.2. Exposure and Outcome Definitions

The primary exposure was the ratio of cumulative installed tower density (towers/km^2^) at the end of the post-epoch (2020) to that at the end of the pre-epoch (2015). Because the principal animal carcinogenicity evidence for RF-EMF used GSM (2G) and CDMA (2G/3G) modulations specifically [23,24], we additionally evaluated the GSM + CDMA + UMTS composite (tower density ratio) as a secondary technology-specific exposure for GBM analyses. Legacy cell tower infrastructure was defined as GSM (2G), a post hoc definition based on falsification checks that GSM passed but the composite and its CDMA and UMTS components failed (Table S1). LTE (4G), which employs fundamentally different modulation (orthogonal frequency-division multiple access; OFDMA) and power management, was evaluated separately. Outcomes were incidence ratios for primary malignant brain tumors, GBM, and VS (included as a negative-control outcome given its distinct histology and exposure geometry relative to glioma). Tower density is an indirect proxy for ambient RF-EMF; OpenCelliD records are cell records classified by their recorded technology and the date first added to the database, not the date of construction, so growth may reflect mapping, with no information on operating status or upgrades. Molecular (IDH) and primary/secondary GBM distinctions were not available or analyzed. Diagnostic methods and confirmation were not available in these data.

### 2.3. Statistical Analysis

Multivariate outliers were identified using Mahalanobis distance computed on z-scored tower density and incidence ratios; counties with distance ≥6 were excluded (6 of 890), leaving 884 counties. To control for pre-existing geographic variation in tumor incidence rates unrelated to cellular infrastructure within our observation period, we adjusted correlations by regressing out baseline (2011–2015) tumor incidence from both the tower density ratios and tumor incidence ratios using ordinary least squares regression. Associations between these residuals were assessed using Pearson correlation tests, two-tailed. Significance was set at *p* < 0.05. Analyses were unweighted, with each county counting equally.

For our analysis of total primary malignant brain tumors, we first performed the same baseline-adjusted analysis as described above. To determine whether these observed correlations were independent of GBM incidence, we performed a further analysis regressing out GBM ratios from both tower density ratios and tumor incidence ratios, again using ordinary least squares regression.

PBT and GBM (with VS as a negative control) were the pre-specified outcomes. Technology-specific, age-stratified, time-window and lag analyses were post hoc and exploratory, with Bonferroni correction applied to four individual technology comparisons (GSM, CDMA, UMTS, LTE) within each outcome, giving *p* < 0.0125 (0.05/4).

### 2.4. Multivariable Confounder Adjustment

To address potential confounding by urbanization, socioeconomic status, healthcare access, and environmental exposures, we constructed a 19-variable confounder model including population and population density (U.S. Census Bureau intercensal county population estimates 2000–2023) and change in percent aged 65 and older between 2011–2015 and 2016–2020 (SEER county population); urbanization (NCHS 6-level urban–rural classification and USDA Beale rural–urban continuum codes); socioeconomic status (median household income, poverty rate, college attainment, uninsured rate, and unemployment rate from the American Community Survey 2016–2020); healthcare access (primary care physicians per 100,000 from County Health Rankings 2020 and Area Health Resources File (AHRF) 2020, hospital beds per 100,000 from Area Health Resources File 2022–2023, short-term general hospitals with MRI per 100,000 from AHRF 2020–2021 (2019 values), and neurosurgeons and diagnostic radiologists per 100,000 from AHRF 2015 (non-federal MDs)); health behavior (adult smoking from County Health Rankings 2016, BRFSS 2014); and environmental exposures (EPA radon zone classification and CDC Tracking PM2.5 highest annual average concentration (monitor + modeled data), averaged over available 2013–2017 values). Of 884 study counties, 865 had complete data for all 19 variables. Of the 19 excluded, 16 lacked primary-care physician data and 3 lacked radon-zone data. For GBM–legacy (GSM), 31 of the 884 counties had no GSM records at the 2015 snapshot, making their density ratios undefined and leaving *n* = 853 for baseline-adjusted analyses. Of those 853, 17 lacked covariate data (14 primary-care physician, 3 radon zone), leaving *n* = 836 for confounder-adjusted analyses. Partial correlations between tower density ratios and tumor incidence ratios were computed within the residuals of both variables after regressing out all confounders and baseline incidence.

Sensitivity analyses used age-standardized rates (Table S2).

We assessed multicollinearity using variance inflation factors (VIFs) in the 19-variable model (Table S3). Residual spatial autocorrelation was assessed using Moran’s I with queen contiguity and 9999 permutations for the baseline-adjusted and 19-variable models.

### 2.5. Repeated-Measures Analyses

To assess robustness using a repeated-measures framework, we conducted a 5-year epoch first-differences analysis treating each county as a two-period panel (pre- and post-epoch), regressing the log change in epoch-averaged incidence on the log change in tower density with an intercept and HC3 95% confidence intervals. This removes all time-invariant county characteristics by construction without requiring their measurement. Baseline-adjusted versions additionally included log baseline (2011–2015) incidence, with and without the 19 covariates. We also estimated a two-way fixed effects model with county and year fixed effects across annual county-years 2010–2021 with county-clustered standard errors.

The main correlations used raw ratios, while the first-difference panels used log ratios. Sensitivity analyses repeated the correlations on the log scale, fitted negative-binomial count models with person-years offsets based on U.S. Census Bureau county population estimates, and weighted the first differences by population (Table S2).

### 2.6. Time Lag and Deployment-Era Sensitivity Analyses

To assess temporal robustness, we conducted a time lag analysis holding the pre-epoch fixed at 2010–2014 and advancing the post-epoch start year, describing how the association varies as temporal separation between exposure and outcome measurement windows increases. The time lag is defined as the difference between the post-epoch and pre-epoch start years. In addition, we conducted a deployment-era sensitivity analysis, varying the pre-epoch start year across the full available range, testing whether the association depends on which deployment era the pre-epoch captures. We evaluated all valid combinations of epoch length {3, 4, 5 years} and start years, yielding 109 specifications. GBM and primary malignant brain tumors vs. all towers were evaluated at each specification; pre-2015 GSM records were too sparse for grid or lag analyses.

In falsification checks, the 2015→2020 tower-density ratio was held fixed and correlated with incidence change in earlier windows ending before the growth it measures (placebo: 2006–2010→2011–2015 and 2001–2005→2006–2010). A significantly positive placebo association would indicate that the correlation reflects stable county characteristics or baseline noise (regression to the mean) rather than a temporally ordered relationship. In the time-reversal check, incidence change was computed backwards (2016–2020→2011–2015), for which a true forward association predicts a non-positive correlation, whereas a baseline-adjustment artifact can reproduce a positive correlation in either direction. The same baseline and 19-variable adjustments were used. An exposure–outcome pairing passed only if the forward association was significantly positive and no placebo or reversed association was significantly positive (Table S1).

### 2.7. Age-Stratified Analyses

To evaluate whether the GBM–tower association varied by age, we stratified incidence counts by the minimum 5-year age group resolution available in SEER for each county and year. We defined three age bands: younger than 55 years, 55–69 years, and 70 years and older. These bands were chosen because GBM incidence rises steeply after age 55, while baseline incidence in the under-55 group is substantially lower (reducing statistical power and the population burden of any association). Baseline-adjusted and confounder-adjusted GBM correlations were repeated within each age band, with rates using band-specific population.

### 2.8. Artificial Intelligence Disclosure

Claude Code (Anthropic) was used to assist with data processing pipeline development, statistical analysis code, and manuscript formatting for this submission. All analyses were verified by the authors, and all scientific conclusions are solely those of the authors.

## 3. Results

### 3.1. Study Population and Geographic Distribution

Our analysis encompassed 1069 counties across 15 states, representing 154,996,485 people, or 46.8% of the 2020 U.S. population; 890 counties remained after excluding counties with zero values in either study period. Multivariate outlier analysis excluded a further 6, leaving 884 counties with a total population of 153,523,984 in 2020 (46.3% of the U.S. population) for final analysis. Cellular infrastructure deployment patterns showed substantial geographic variation in growth rates between pre-inflection (2011–2015) and post-inflection (2016–2020) epochs (Figure 1B) with a coefficient of variation (CoV) of 0.34. Each generation type showed substantial deployment between our study epochs, except for NR (5G) technology, which only began deployment in 2020 and was thus excluded from our analysis.

### 3.2. Association Between Cellular Infrastructure Deployment and Primary Malignant Brain Tumors

**Figure 2:**
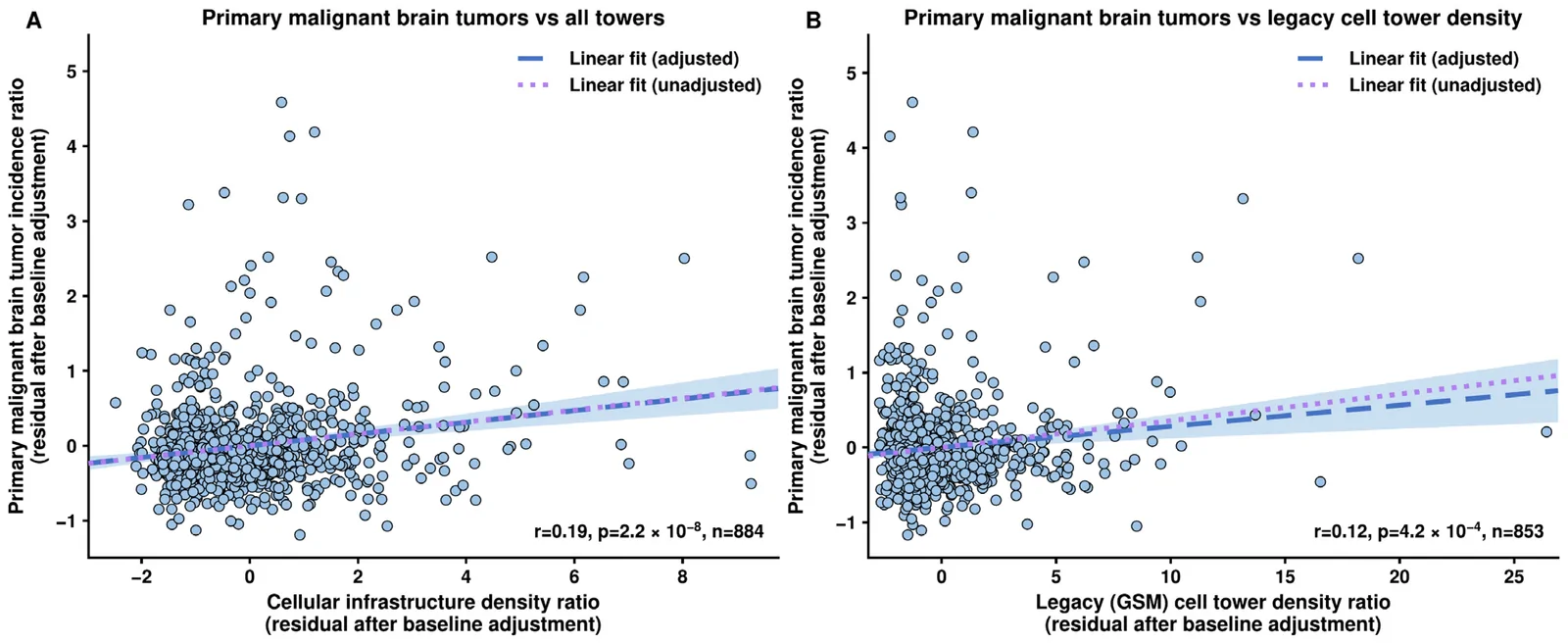
Cellular infrastructure association with primary malignant brain tumor incidence. (**A**) Scatter plot showing the relationship between county tumor incidence ratios and all-tower infrastructure density ratios, both residualized for baseline (2011–2015) incidence. The blue dashed line and shaded area represent a regression fit with a 95% confidence interval; the purple dotted line shows the unadjusted regression for comparison. Overall cellular infrastructure density increases show a modest but significant correlation with total primary malignant brain tumor incidence increase (r = 0.19, *p* = 2.2 × 10^−8^; unadjusted r = 0.16, *p* = 1.0 × 10^−6^). (**B**) Corresponding scatter plot for legacy (GSM) cell tower infrastructure density ratios (r = 0.121, *p* = 4.2 × 10^−4^; full technology breakdown in Supplementary Table S4). The blue dashed line and shaded area represent a regression fit with a 95% confidence interval; the purple dotted line shows the unadjusted regression for comparison.

Our analysis of primary malignant brain tumors showed substantial variation in post-inflection to pre-inflection incidence ratios between counties (CoV = 0.62; Figure 1D). We found a statistically significant correlation between total incidence ratios and cellular infrastructure density ratios (r = 0.16, *p* = 1.0 × 10^−6^, 95% CI 0.099 to 0.227, *n* = 884; Figure 2A, purple dotted line). This association remained significant after controlling for baseline incidence (r = 0.19, *p* = 2.2 × 10^−8^, 95% CI 0.122 to 0.250, blue dashed line), as well as for GBM incidence ratios (r = 0.18, *p* = 4.0 × 10^−8^, 95% CI 0.119 to 0.247, not displayed), while changes in total primary malignant brain tumors excluding GBMs also correlated significantly with cellular infrastructure density (r = 0.12, *p* = 1.1 × 10^−3^, 95% CI 0.047 to 0.186, unadjusted r = 0.13, *p* = 4.5 × 10^−4^, 95% CI 0.056 to 0.195, Figure S1). After full 19-variable confounder adjustment (*n* = 865 counties), the association remained significant (r = 0.12, *p* = 4.4 × 10^−4^, 95% CI 0.054 to 0.187; Figure S2), but the non-GBM component attenuated to non-significance (r = 0.033, *p* = 0.38, 95% CI −0.040 to 0.104), indicating that the confounder-adjusted total primary malignant brain tumor and GBM associations remained significant.

Legacy cell tower infrastructure density (GSM, defined post hoc) showed a significant baseline-adjusted association with primary malignant brain tumor incidence ratios (r = 0.121, *p* = 4.2 × 10^−4^, 95% CI 0.054 to 0.186; Figure 2B). Technology-specific associations for total primary malignant brain tumors were broadly positive across technology subtypes (Supplementary Table S4); however, after 19-variable confounder adjustment, only the 2G/3G composite (r = 0.094, *p* = 0.0062, 95% CI 0.027 to 0.161) and GSM specifically (r = 0.125, *p* = 3.4 × 10^−4^, 95% CI 0.057 to 0.192) remained significant. Both GSM and the composite met the Bonferroni threshold (*p* < 0.0125), but the composite failed falsification checks under both adjustments for PBT and GBM (Table S1). CDMA, UMTS, and LTE were non-significant after confounder adjustment.

### 3.3. Association Between Cellular Infrastructure Deployment and GBM Incidence

**Figure 3:**
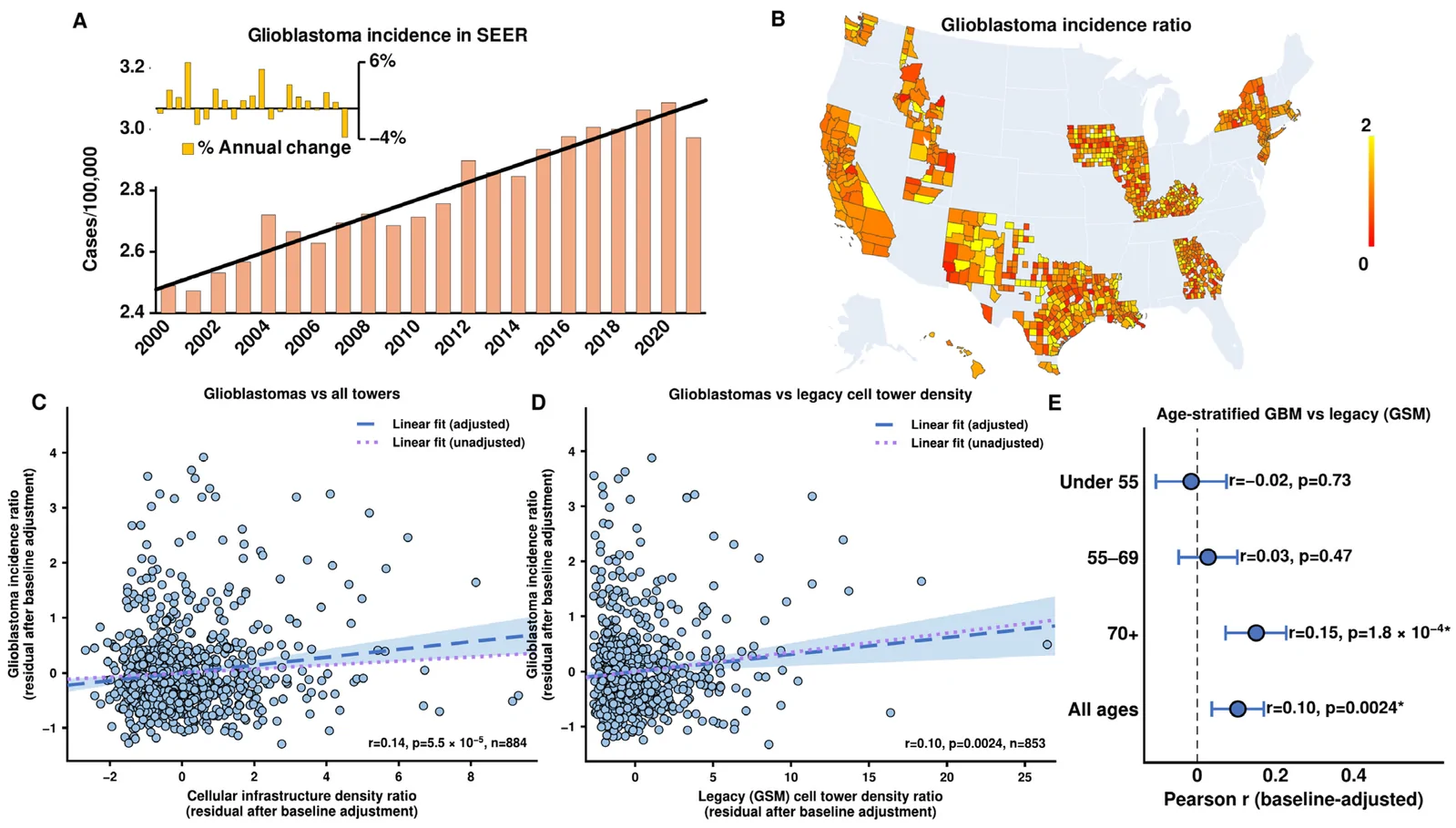
Cellular infrastructure association with glioblastoma incidence. (**A**) Annual total GBM incidence per 100,000 people across counties in the most recent SEER county-level data available (2000–2021) shows an annual increase of 1.0% (black trendline). (**B**) Corresponding geographic variation in GBM incidence growth, shown as the ratio of average cases per 100,000 people between epochs. (**C**) Scatter plot showing the relationship between county GBM incidence ratios and all-tower infrastructure density ratios, both residualized for baseline (2011–2015) incidence. Overall cellular infrastructure density increases show a significant correlation with GBMs (r = 0.14, *p* = 5.5 × 10^−5^; unadjusted r = 0.061, *p* = 0.069). (**D**) Corresponding scatter for legacy (GSM) cell tower infrastructure density ratios (r = 0.104, *p* = 0.0024). The blue dashed line and shaded area represent a regression fit with a 95% confidence interval; the purple dotted line shows the unadjusted regression for comparison. (**E**) Forest plot of baseline-adjusted Pearson r with 95% confidence intervals for GBM vs. legacy (GSM) cell tower density ratios stratified by age band (under 55, 55–69, and 70+ years), alongside the all-age reference. Rates use band-specific person-years. Asterisks indicate significance at *p* < 0.05. Confounder-adjusted estimates across age bands are reported in Supplementary Table S5.

In line with previous reports [18], GBM incidence within our study period increased by 1.0% annually in SEER counties (Figure 3A). Changes in GBM incidence demonstrated similar geographic variation to cellular infrastructure growth (CoV = 0.68, Figure 3B). After controlling for baseline incidence, changes in overall cellular infrastructure density showed a significant positive correlation with changes in GBM incidence (r = 0.14, *p* = 5.5 × 10^−5^, 95% CI 0.070 to 0.200, *n* = 884, blue dashed line; unadjusted r = 0.061, *p* = 0.069, 95% CI −0.005 to 0.127, purple dotted line, Figure 3C). The association remained significant after full 19-variable confounder adjustment (r = 0.083, *p* = 0.016, 95% CI 0.016 to 0.150).

Technology-specific analyses revealed a variable pattern of associations with cell tower generation (Supplementary Table S4). The association with legacy cell tower infrastructure was significant (r = 0.104, *p* = 0.0024, 95% CI 0.037 to 0.170; Figure 3D). This post hoc GSM association was model-dependent (Table 1 and Table S2). LTE (4G) showed no significant association (r = 0.052, *p* = 0.12, 95% CI −0.014 to 0.118). After 19-variable confounder adjustment, within the GSM + CDMA + UMTS composite, only GSM remained significant after Bonferroni correction (r = 0.110, *p* = 0.0017, 95% CI 0.041 to 0.177); CDMA and UMTS attenuated to non-significance (Supplementary Table S4). The GSM + CDMA + UMTS composite had a significant correlation (r = 0.124, *p* = 3.2 × 10^−4^, 95% CI 0.057 to 0.189; Supplementary Table S4) but failed falsification checks under both adjustments for GBM and PBT (Table S1).

In the all-towers model, VIFs were 16.1 and 16.4 for the CHR and AHRF primary-care physician measures (r = 0.97), 8.2 and 7.3 for NCHS urban–rural and Beale codes (r = 0.92), and 6.2 for median income; all other covariate VIFs were <4.7, with an exposure-ratio VIF of 1.2 and baseline-incidence VIF of 1.1. The legacy exposure-ratio VIF was 1.0; Table S3 reports VIFs for each model. Collinearity was concentrated among paired covariates measuring the same construct, rather than between exposure and covariates.

Residual Moran’s I for baseline-adjusted PBT–all, GBM–all and GBM–legacy models was 0.035 (*p* = 0.064), 0.058 (*p* = 0.007) and 0.057 (*p* = 0.011), respectively; after 19-variable adjustment, corresponding values were 0.004 (*p* = 0.40), 0.032 (*p* = 0.09) and 0.022 (*p* = 0.17). Residual spatial autocorrelation was small and detectable for GBM in baseline-only models, but not after confounder adjustment.

### 3.4. Age-Stratified Analyses

In the age-stratified analysis of GBM vs. legacy cell tower density, the association was not significant in adults younger than 55 years (*n* = 477 counties with sufficient case counts in both epochs; baseline-adjusted r = −0.016, *p* = 0.73, 95% CI −0.105 to 0.074; Figure 3E) or after 19-variable confounder adjustment (r = −0.034, *p* = 0.47, 95% CI −0.125 to 0.058). Associations were also non-significant in adults aged 55–69 years (*n* = 684 counties; r = 0.028, *p* = 0.47, 95% CI −0.047 to 0.102; confounder-adjusted r = 0.025, *p* = 0.53, 95% CI −0.052 to 0.101), but significant in adults aged 70 years and older (*n* = 614 counties; r = 0.151, *p* = 1.8 × 10^−4^, 95% CI 0.072 to 0.227; confounder-adjusted r = 0.137, *p* = 9.1 × 10^−4^, 95% CI 0.056 to 0.215; Supplementary Table S5). LTE associations were non-significant within each age band (Supplementary Table S5).

### 3.5. Repeated-Measures Analyses

A 5-year epoch first-differences panel analysis, which removes all time-invariant county-level characteristics by construction, included an intercept and yielded an unadjusted GBM–all coefficient of 0.034 (95% CI −0.120 to 0.187, *p* = 0.67, *n* = 884) and a baseline-adjusted coefficient of 0.125 (95% CI −0.001 to 0.251, *p* = 0.052); corresponding GBM–legacy coefficients were 0.055 (95% CI −0.036 to 0.145, *p* = 0.24) and 0.014 (95% CI −0.060 to 0.088, *p* = 0.70). Primary malignant brain tumors had an unadjusted coefficient of 0.167 (95% CI 0.026 to 0.308, *p* = 0.021) and a baseline-adjusted coefficient of 0.107 (95% CI −0.000 to 0.214, *p* = 0.050). A two-way fixed effects model (county + year fixed effects, annual panel 2010–2021) yielded non-significant results for all tumor types vs. all towers (GBM: coef = −0.012, *p* = 0.46, 95% CI −0.044 to 0.020). The narrow GBM–all confidence interval provides an informative null for the annual within-county association. Tower growth was positively correlated with baseline incidence, while incidence ratios were negatively correlated with baseline incidence (regression to the mean), which may account for the difference between unadjusted and baseline-adjusted first-difference estimates.

All-tower log correlations and negative-binomial count models adjusted for baseline incidence were significantly positive; negative-binomial count models adjusted for baseline incidence plus the 19 confounders were non-significant, while age-standardization retained significant correlations (Table S2). Positive GSM correlations were unsupported by log-scale, count or first-difference models; fixed-effects estimates were negative (Table 1 and Table S2).

### 3.6. Temporal Robustness

**Figure 4:**
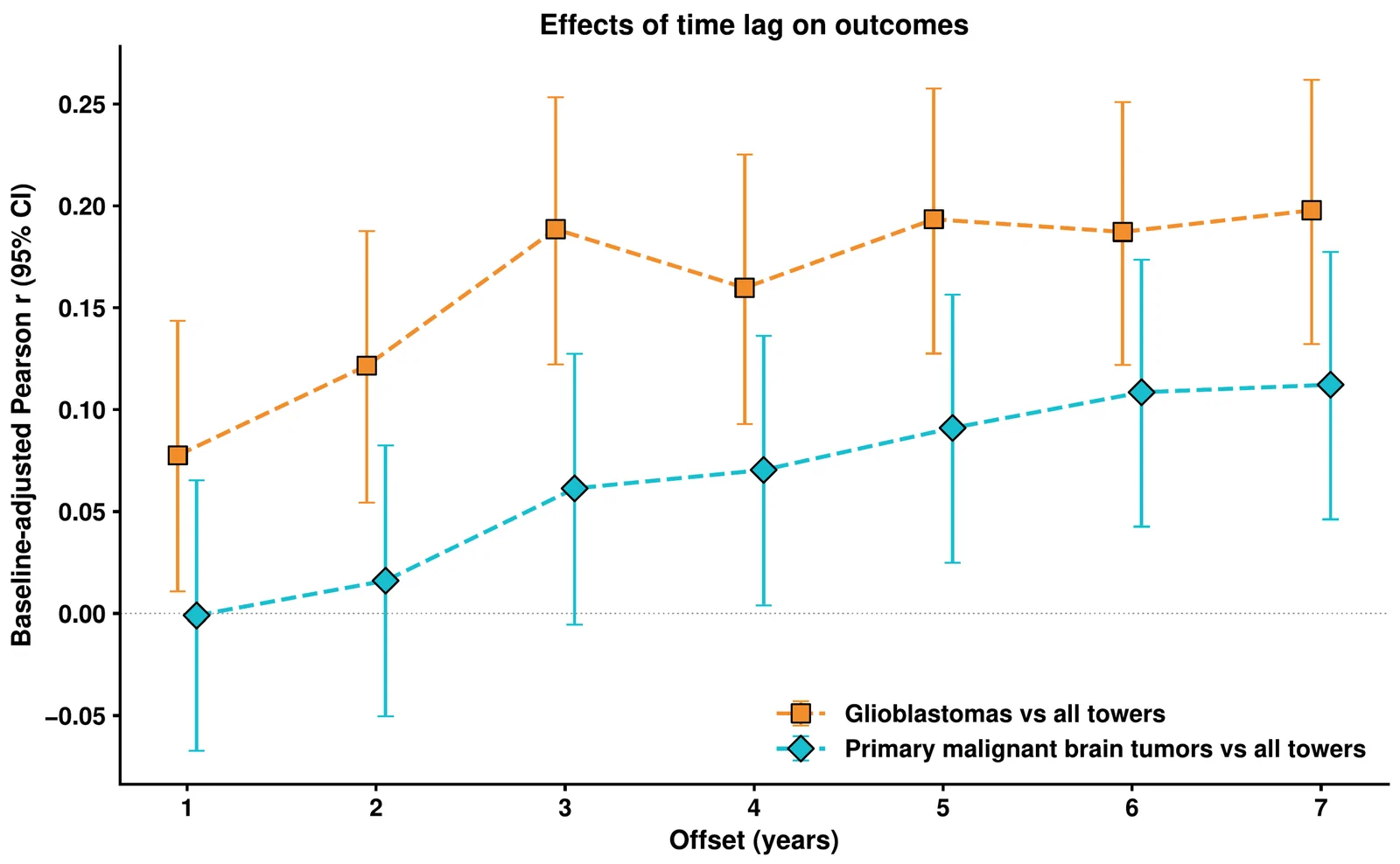
Time lag analysis (pre-epoch fixed; post-epoch advancing). Baseline-adjusted Pearson r (with 95% confidence intervals) for two outcome–exposure pairings: GBM vs. all towers and primary malignant brain tumors (PBT) vs. all towers as a function of lag between post-epoch and pre-epoch, with pre-epoch fixed at 2010–2014 and post-epoch start year ranging from 2011 to 2017 (lag = 1 to 7).

Time lag analysis showed variation in associations as the temporal separation between epochs increased (Figure 4). With the pre-epoch fixed at 2010–2014, the GBM vs. all-towers association varied from r = 0.078 at lag = 1 to r = 0.198 at lag = 7 (full results in Figure 4). Lags 1–4 use overlapping windows, and secular or other time-related factors could produce this pattern.

The deployment-era sensitivity analysis (Figure S3) tested whether the association depends on which deployment era the pre-epoch captures. Across the 109 specifications for GBM vs. all towers, 52 of 78 specifications with pre-epoch start ≤2013 were significant and positive. For pre-epoch start 2014–2015, none of 21 specifications were significant. For pre-epoch start ≥2016, none of 10 specifications were significant; no specification was significant in the negative direction.

In placebo and time-reversal falsification checks, PBT–all and GSM pairings passed under both adjustments, GBM–all passed with full adjustment, and the GSM + CDMA + UMTS composite, CDMA and UMTS failed (Table S1).

### 3.7. No Significant Association Between Cellular Infrastructure Deployment and VS

VS showed an average annual increase of 5.6% with 647 counties included in our final analysis (Figure S4A). Post-inflection to pre-inflection incidence ratios showed considerable variation (CoV = 0.81, Figure S4B). Analysis revealed no significant association between cellular infrastructure growth and VS incidence (baseline-adjusted r = 0.045, *p* = 0.25, 95% CI −0.032 to 0.122, blue dashed line and unadjusted r = 0.028, *p* = 0.48, 95% CI −0.049 to 0.105, purple dotted line, Figure S4C). This non-significant result persisted after 19-variable confounder adjustment (confounder-adjusted r = 0.024, *p* = 0.55, 95% CI −0.055 to 0.102).

A secondary analysis against legacy cell tower infrastructure showed no significant association (baseline-adjusted r = 0.012, *p* = 0.75, 95% CI −0.065 to 0.090), including after 19-variable confounder adjustment (r = 0.016, *p* = 0.70, 95% CI −0.063 to 0.094). This is consistent with the non-significant result for VS overall: the legacy signal does not parallel the significant GBM–legacy correlation.

## 4. Discussion

Our population-based study found that county-level growth in cellular infrastructure density was associated with growth in total primary malignant brain tumor incidence, including GBM after confounder adjustment. This association with GBM incidence was significant for legacy cellular infrastructure and non-significant for LTE and was present only in the oldest age band. The non-GBM component of the association attenuated after confounder adjustment, and the VS negative control showed no significant association. LTE, non-GBM after full confounder adjustment, all-tower two-way fixed-effects models, and unadjusted first-difference models for GBM yielded non-significant results.

### 4.1. Biological Effects of RF-EMF Exposure

The potential health effects of RF-EMF have been studied through both laboratory and clinical research. The National Toxicology Program provided evidence for biological effects that may contribute to carcinogenesis, with rats exposed to RF-EMF within the GSM (2G) and CDMA (2G/3G) spectra showing increased incidence of heart schwannomas and malignant glial tumors at the highest levels of exposure [23,24,25]. Notably, evidence suggests RF-EMF appears to act as a tumor-promoting cocarcinogen [26,27,28] by inducing DNA damage in both mouse embryonic fibroblasts [29] and human ear canal hair follicle cells, even demonstrating a potential dose-dependent effect [30].

Studies of RF-EMF absorption patterns have revealed potential mechanisms of harm. The vast majority of RF-EMF is absorbed in the hemisphere on the side of phone use, with substantial concentration in the temporal lobe. Absorption of RF-EMF from cell phones drops off rapidly with depth, particularly at higher transmission frequencies [31]. These effects have been quantified through measurements of altered cerebral blood flow in healthy volunteers, particularly within the inferior temporal and prefrontal cortex [9,32,33], as well as increased intracortical excitability [34] and increased alpha-band power on EEG [32]. Metabolic PET has demonstrated increased glucose uptake in regions closest to the antenna, particularly the temporal pole and orbitofrontal cortex [8]. These physiological changes raise particular concern for the pediatric age group, with anatomical modeling showing that children’s brains absorb substantially higher local radiation doses than adults’ brains [35]. While not direct evidence of carcinogenesis, these studies demonstrate that RF-EMF can alter biology in dose-dependent and spatially specific ways.

### 4.2. Prior Epidemiological Evidence

The INTERPHONE study, a large multinational case–control investigation, found there might be an elevated risk for glioma and meningioma among the heaviest mobile phone users, with higher risks observed in the temporal lobe and on the same side as reported phone use [6]. While the authors caution that these findings are exploratory, subject to recall biases, and fail to show causation, survey-based methods appear to provide a fair estimate of the amount and laterality of mobile phone use [36], particularly given that the effect of random errors leads to under-estimation rather than over-estimation of risk [37]. However, the study found no overall increased risk for routine users.

The Danish nationwide register-based cohort study found slightly increased but non-significant risks for glioma among male subscribers, with a modest elevation in temporal lobe gliomas specifically [38]. The study found no increased risk for meningiomas or overall CNS tumors, and no dose–response relationship with subscription length. While this study contrasted subscribers with non-subscribers, it did not attempt to quantify individual mobile phone usage and its methodology has been challenged [4,39,40].

The Swedish case–control studies provided strong evidence for long-term effects, demonstrating a clear dose-dependent relationship between mobile phone use and glioma risk that increased substantially with longer exposure periods, with the highest risk for ipsilateral temporal lobe glioma [41]. The risk was particularly elevated for users who began before age 20. Further analysis revealed that long-term mobile phone use was associated with decreased survival in glioma patients in a significantly dose-dependent manner, with an even stronger effect specifically for GBM. These studies derive from a single research group using hospital-based controls, and the possibility of selection and recall bias has been documented.

Research on VS has shown particularly strong associations with older technology, with the Swedish studies showing strongly increased risk with longer latency periods and a significant dose-dependent relationship on the ipsilateral side [42]. A South Korean case–control study demonstrated clear correlations between tumor volume and estimated ipsilateral cumulative phone use [43]. While we found no significant correlation with changes in VS incidence in our cohort, technology-specific analysis showed a similar pattern to previous studies with a positive association with some older generations, including UMTS, but not GSM or CDMA (Supplementary Table S4).

More recent large-scale studies have produced mixed results. COSMOS, the largest prospective cohort study to date, showed no increased risk with cumulative cell phone use [44]. This well-designed prospective study avoids recall bias by collecting exposure data before diagnosis. Methodological challenges include the difficulty of defining an unexposed reference group given near-universal phone adoption [40], reliance on baseline exposure assessment in a rapidly changing technology landscape, and follow-up that may not capture long-latency effects [45]. Our study examines county-level infrastructure density across technology generations in exploratory, post hoc analyses. Additionally, while COSMOS relied on baseline exposure data, our study captures dynamic changes in exposure patterns through temporal analysis of infrastructure deployment, allowing us to examine how changes in environmental RF-EMF relate to tumor incidence. Finally, by analyzing counties that were geographically dispersed and representing almost half of the U.S. population, our study achieved sufficient statistical power to detect subtle associations that might be missed in smaller-scale studies.

Notably, several recent systematic reviews have demonstrated significant associations between long-term, cumulative mobile phone exposure and glioma risk on the ipsilateral side [2,46]. The WHO’s comprehensive meta-review across numerous countries found no elevated overall risk for glioma, meningioma, or VS, but did reveal an increasing trend for glioma risk with cumulative use [47].

### 4.3. Prior Ecological Analyses

Prior ecological and time-trend analyses have examined population-level brain tumor rates in relation to mobile phone adoption. Nordic analyses found stable overall brain tumor incidence despite rapidly increasing mobile phone use [15]. Australian analyses spanning nearly three decades found no increase in brain tumor rates [16]. In the UK, Bayesian time-series analyses found limited evidence of a mobile phone effect on brain tumor subtypes, though rising GBM trends have been documented there [17]. US registry data have similarly shown GBM increases alongside stable overall brain tumor rates [18], with the cause remaining incompletely explained.

These prior ecological analyses typically examined national or regional aggregate trends using subscription rates or adoption metrics as exposure proxies. Our study differs in using county-level infrastructure density—a geographically specific proxy for ambient exposure—and in analyzing temporal growth rates rather than absolute levels, which reduces confounding by stable geographic differences. The technology-generation stratification, multivariable confounder adjustment, repeated-measures analyses, and age-stratified analyses represent methodological additions relative to prior ecological work in this area.

Ecological analyses occupy a specific position in the epidemiological evidence hierarchy. They can identify population-level patterns and generate hypotheses about exposures affecting large populations, but they cannot establish individual-level risk because county-level correlations do not demonstrate that the most-exposed individuals are those developing tumors, a limitation known as the ecological fallacy. Case–control and prospective cohort studies are better suited to individual-level inference but face their own challenges in the RF-EMF context: recall bias in retrospective designs, the difficulty of defining an unexposed reference group as phone use approaches saturation, and follow-up periods that may be shorter than tumor latency. Our findings complement rather than supersede these individual-level designs.

### 4.4. Present Findings

Our analysis of cellular infrastructure density as an exposure proxy avoids the recall bias and measurement error associated with self-reported mobile phone use. While existing literature has focused on personal RF-EMF exposure from mobile devices, our study is among the first US population-scale analyses of ambient RF-EMF exposure from cellular infrastructure and primary malignant brain tumor incidence. Unlike the high-intensity but episodic exposure patterns associated with active cell phone use, cell towers generate continuous, involuntary exposure with considerable spatial variability based on proximity to antenna installations—a distinct exposure pattern that has received less epidemiological attention [11].

Cellular tower density was associated with primary malignant brain tumor incidence increase. This effect size is modest. The rapid proliferation of RF-EMF sources in the modern environment, particularly the ongoing deployment of 5G infrastructure, suggests that continued surveillance is warranted regardless of the modest per-county effect size [48,49,50].

#### 4.4.1. Technology-Specific Associations

The technical characteristics of radiofrequency exposure vary across cellular generations. Earlier technologies like GSM (2G) operate in the 850–1900 MHz range with pulsed emissions and relatively high peak power during voice transmission, while CDMA-based technologies (2G/3G) and UMTS (3G) use spread spectrum techniques, leading to more continuous emissions and dynamic power control. Newer technologies like LTE (4G) and beyond incorporate more advanced power management systems with improved efficiency. The principal animal carcinogenicity studies demonstrating malignant glial tumors and schwannomas used GSM and CDMA modulations specifically [23,24].

While our analysis revealed modest positive correlations between changes in each technology type and primary malignant brain tumor incidence, the associations with GBM and VS incidence were less consistent and were non-significant for LTE. The association with GBM was significant for legacy cell tower infrastructure (r = 0.104, *p* = 0.0024), while LTE showed no significant association (r = 0.052, *p* = 0.12). The contrast between the legacy cell tower and LTE findings is descriptive. LTE tower deployment and legacy cell tower deployment occurred at different times; the difference between correlations was not formally tested and does not rule out confounding.

#### 4.4.2. Age-Stratified Findings

Recent tower-density growth is interpreted here as an indirect proxy for county-level RF infrastructure burden, not as a claim that 2011–2020 exposure alone caused tumors diagnosed during that interval. Older adults had higher baseline GBM risk, whereas younger adults had substantially lower incidence during the study window. The GBM–legacy cell tower association was not significant in adults younger than 55 (baseline-adjusted r = −0.016, *p* = 0.73; confounder-adjusted r = −0.034, *p* = 0.47) or those aged 55–69 (adjusted r = 0.025, *p* = 0.53), and was significant in those aged 70 and older (adjusted r = 0.137, *p* = 9.1 × 10^−4^). The age pattern could also reflect age-specific diagnostic intensity or referral, which could not be measured in these data. LTE associations were non-significant within each age band.

#### 4.4.3. Contextual Interpretation

FCC policies aimed at expanding rural internet access [51] led to markedly different rates of cellular infrastructure growth across counties during our study period. This variation in infrastructure deployment provided an opportunity to examine whether regions with greater increases in cellular tower density showed corresponding changes in primary malignant brain tumor incidence. We observed steady annual increases in GBM incidence across SEER counties, aligning with trends seen in other studies [18] and in other developed countries [17].

The relationship with total primary malignant brain tumors independent of GBMs attenuated to non-significance after full 19-variable confounder adjustment, without establishing absence of an association for other tumor types. Case–control studies have shown stronger associations for gliomas compared to other brain tumors, but these include low-grade gliomas as well [7].

Our VS findings present a contrast with the phone use literature. While multiple case–control studies have shown associations between phone use and VS [42,43], we found no significant association with infrastructure density. Against legacy cell tower infrastructure, the association was non-significant both before (baseline-adjusted r = 0.012, *p* = 0.75) and after 19-variable confounder adjustment (r = 0.016, *p* = 0.70). VS is an imperfect negative control because exposure geometry, incidence, ascertainment and statistical power differ between VS and GBM; its non-significant result does not establish absence of confounding. In addition, evidence from case–control studies has been strongest for glioma, and our analysis may not be sensitive enough to detect weaker correlations with other tumor types.

### 4.5. Limitations and Future Directions

Our study demonstrates a modest association between cellular infrastructure density and total primary malignant brain tumor incidence, as well as GBM incidence, but all-tower associations were non-significant in the negative-binomial count model adjusted for baseline incidence plus all 19 confounders, the first-difference model adjusted for baseline incidence plus the confounders, and the two-way fixed-effects model (Table 1 and Table S2). The epidemiological study design, though allowing examination of population-level trends, cannot definitively attribute these associations to RF-EMF exposure versus underlying factors that might drive infrastructure deployment. As an ecological study, our analysis is subject to the ecological fallacy, in that county-level correlations between infrastructure density and tumor incidence do not establish that individuals with the greatest RF-EMF exposure are those developing tumors. The observed association could reflect any county-level factor correlated with both tower growth and tumor ascertainment.

Tower density is a crude proxy for individual RF-EMF dose. It does not account for transmission power, distance from towers, indoor versus outdoor exposure, antenna directionality, frequency, building penetration, terrain effects, or concurrent personal device use. Counties with similar tower density may have very different actual ambient field strengths. Tower density may instead track urbanization, telecommunications demand, population growth or mapping activity. Future research should examine exposure patterns integrating both ambient infrastructure exposure and personal device use [36].

External validation of OpenCelliD dates was not possible: OpenCelliD is the largest open database of its kind, and no publicly available source provides comparable dated, technology-specific cell-site records. FCC Antenna Structure Registration, the closest candidate, covers only registered tall or airport-adjacent structures of any use and records no technology. In the 884 study counties in 2020, OpenCelliD held about 3.1 million cell records versus about 50,000 FCC-registered antenna structures of any use (roughly 60-fold).

Despite the 19-variable confounder model, residual confounding remains possible. Urbanization drives both infrastructure deployment and diagnostic intensity (including MRI availability and neurosurgical referral patterns), creating a well-recognized confounding pathway. Our model explicitly includes urbanization, healthcare access (including hospital MRI availability and diagnostic radiologist and neurosurgeon densities), socioeconomic indicators and adult smoking, but individual MRI use and referral patterns, occupational exposures, ionizing radiation, genetic/familial predisposition and healthcare-seeking behavior remain unmeasured. For the age-stratified analyses specifically, age-specific patterns in diagnostic access, such as differential MRI rates or neurosurgical referral for older versus younger adults, represent potential sources of residual confounding that the model does not fully address. Residual spatial autocorrelation detectable in the baseline-only GBM models means their *p*-values may be somewhat optimistic; it was not detectable after confounder adjustment.

The time lag analysis covers separations of up to 7 years between epoch start years. Additionally, our time window may not fully capture latency periods that some studies suggest could exceed two decades [7].

Primary analyses weighted each county equally, and small-county incidence rates may be unstable. Changes in county age composition between epochs are not addressed by the crude rates alone (Table S2). Noisy baselines can bias adjusted associations through regression to the mean. The association with the post hoc GSM definition is model-dependent. Bonferroni correction does not address overall multiplicity. Exclusion of counties with zero GBM cases and of outliers may introduce selection bias. Incomplete SEER geographic coverage limits generalizability.

These findings are hypothesis-generating and require confirmation through mechanistic and individual-level studies. As 5G infrastructure continues to deploy, sustained population-level surveillance combined with dosimetric and mechanistic research will be essential to clarifying whether the patterns observed here reflect a real exposure-disease relationship.

## 5. Conclusions

County-level growth of cellular infrastructure is modestly associated with primary malignant brain tumor incidence. The legacy association was significant only in adults aged 70 and older; LTE and vestibular schwannoma showed no significant association. These findings are hypothesis-generating; causation is not established.

## Figures and Tables

**Table 1:** Comparison of unadjusted, baseline-adjusted and 19-variable-adjusted correlations; first-difference models (unadjusted, + baseline incidence, and + baseline incidence + 19 variables); and two-way fixed-effects models for PBT–all, GBM–all and GBM–legacy (GSM). Estimates are Pearson r for correlation models and log-scale regression coefficients for first-difference and fixed-effects models, with 95% CIs, *p*-values and sample sizes (*n* denotes counties except county-years for the fixed-effects row). First-difference models include an intercept and use HC3 CIs; two-way fixed-effects models include county and year effects with county-clustered uncertainty.

Model	Scale	PBT vs. All Towers	GBM vs. All Towers	GBM vs. Legacy (GSM)
Unadjusted correlation	r	0.163 (0.099 to 0.227); *p* = 1.0 × 10^−6^; *n* = 884	0.061 (−0.005 to 0.127); *p* = 0.069; *n* = 884	0.102 (0.035 to 0.168); *p* = 0.003; *n* = 853
Baseline-adjusted correlation	partial r	0.187 (0.122 to 0.250); *p* = 2.2 × 10^−8^; *n* = 884	0.135 (0.070 to 0.200); *p* = 5.5 × 10^−5^; *n* = 884	0.104 (0.037 to 0.170); *p* = 0.002; *n* = 853
19-variable adjusted correlation	partial r	0.121 (0.054 to 0.187); *p* = 4.4 × 10^−4^; *n* = 865	0.083 (0.016 to 0.150); *p* = 0.016; *n* = 865	0.110 (0.041 to 0.177); *p* = 0.002; *n* = 836
First difference (log scale)	slope	0.167 (0.026 to 0.308); *p* = 0.021; *n* = 884	0.034 (−0.120 to 0.187); *p* = 0.67; *n* = 884	0.055 (−0.036 to 0.145); *p* = 0.24; *n* = 853
First difference + baseline incidence	slope	0.107 (0.000 to 0.214); *p* = 0.050; *n* = 884	0.125 (−0.001 to 0.251); *p* = 0.052; *n* = 884	0.014 (−0.060 to 0.088); *p* = 0.70; *n* = 853
First difference + baseline + 19 variables	slope	0.057 (−0.055 to 0.170); *p* = 0.32; *n* = 865	0.106 (−0.031 to 0.243); *p* = 0.13; *n* = 865	0.036 (−0.037 to 0.109); *p* = 0.33; *n* = 836
Two-way fixed effects (annual, 2010–2021)	slope	−0.002 (−0.029 to 0.026); *p* = 0.90; *n* = 8225 county-years	−0.012 (−0.044 to 0.020); *p* = 0.46; *n* = 7021 county-years	−0.036 (−0.070 to −0.001); *p* = 0.041; *n* = 5010 county-years

## Data Availability

The data underlying this article are available in Zenodo (https://doi.org/10.5281/zenodo.23522237, accessed on 19 October 2026). The datasets were derived from the following sources, all publicly available except SEER Research Plus data, which are available from the SEER Program under a data use agreement: (1) The National Cancer Institute’s Surveillance, Epidemiology, and End Results (SEER) Program Research Plus Limited-Field Data, 22 Registries, Nov 2023 Sub (2000–2021) database (https://www.seer.cancer.gov/, accessed on 19 October 2026). (2) OpenCelliD cellular infrastructure database (https://www.opencellid.org/, accessed on 19 October 2026). (3) U.S. Census Bureau intercensal county population estimates (2000–2023) (https://www.census.gov/data/tables/time-series/demo/popest/intercensal-2000–2010-counties.html, https://www2.census.gov/programs-surveys/popest/datasets/2010-2019/counties/totals/, and https://www.census.gov/data/tables/time-series/demo/popest/2020s-counties-total.html, accessed on 19 October 2026). (4) U.S. Census Bureau TIGER/Line county boundary shape files (https://www2.census.gov/geo/tiger/TIGER2023/STATE/, accessed on 19 October 2026). (5) NCHS Urban–Rural Classification Scheme for Counties (2013) (https://www.cdc.gov/nchs/data-analysis-tools/urban-rural.html, accessed on 19 October 2026). (6) U.S. Census Bureau American Community Survey 5-year estimates (2016–2020) (https://www.census.gov/data/developers/data-sets/acs-5year.html, accessed on 19 October 2026). (7) Health Resources and Services Administration Area Health Resources File (AHRF) 2022–2023. USDA Beale Rural–Urban Continuum Codes are included in this release (https://data.hrsa.gov/topics/health-workforce/ahrf, accessed on 19 October 2026). (8) County Health Rankings & Roadmaps 2020 (https://www.countyhealthrankings.org/health-data/methodology-and-sources/data-documentation, accessed on 19 October 2026). (9) U.S. Environmental Protection Agency Map of Radon Zones (https://www.epa.gov/radon/epa-map-radon-zones, accessed on 19 October 2026). (10) U.S. Environmental Protection Agency modeled county-level PM2.5 annual mean concentrations (https://ephtracking.cdc.gov/DataExplorer/, accessed on 19 October 2026). The data analysis scripts that generated the results will also be made available in Zenodo.

## Supplementary Materials

The following supporting information can be downloaded at https://www.mdpi.com/article/10.3390/cancers18213419/s1. Supplementary Methods; Figure S1: Primary malignant brain tumors excluding GBM; Figure S2: Confounder-adjusted associations (forest plot); Figure S3: Deployment-era analysis (pre-epoch era varying); Figure S4: No significant association between cellular infrastructure growth and vestibular schwannoma incidence; Table S1: Placebo and time-reversal falsification analyses; Table S2: Sensitivity analyses for PBT–all towers, GBM–all towers and GBM–legacy (GSM); Table S3: Variance inflation factors (VIFs) for the 19-variable model, including the exposure ratio and baseline incidence; Table S4: Confounder-adjusted Pearson’s correlations between primary malignant brain tumor, glioblastoma (GBM), and vestibular schwannoma (VS) incidence ratios and cellular infrastructure density ratios, for the GSM + CDMA + UMTS composite as well as each tower subtype; Table S5: Age-stratified GBM results. References [19,20,21,22,52,53,54,55,56,57,58,59,60,61] are cited in the Supplementary Materials.
