# Supplementary material for: Ecological Analysis of Spatiotemporal Patterns in Brain Tumor Incidence and Cellular Infrastructure Growth in the United States
Source: Cancers (Basel). 2026 Oct 22;18(21):3419. doi: 10.3390/cancers18213419 (PMC13762011; doi:10.3390/cancers18213419)
Supplement: Supplementary file 1 [file cancers-18-03419-s001.zip › cancers-4639295-supplementary.pdf]

Supplementary Figure and Table Legends

Table S1: Placebo and time-reversal falsification analyses. Exposure is held fixed at the 2015→2020 density ratio. Forward incidence change is 2011–2015→2016–2020; placebo A is 2006–2010→2011–2015; placebo B is 2001–2005→2006–2010; reversal is 2016–2020→2011–2015. The same correlation procedure is applied with baseline and 19-variable adjustment; population denominators use the master table for 2010–2020 and SEER county population for 2001–2009. PASS requires a significantly positive forward association and no significantly positive placebo or reversal association (two-sided p<0.05); a temporally directional effect predicts a non-positive reversal correlation. Pearson r and p-values are shown for each run. These checks do not establish causality.

Association	Adjustment	2011–15 → 2016–20 (forward)	Placebo 2006–10 → 2011–15	Placebo 2001–05 → 2006–10	Reversed 2016–20 → 2011–15	Gate
PBT vs all towers	Baseline	0.187 (p=2.2 × 10^{-8})	0.009 (p=0.79)	0.060 (p=0.076)	0.035 (p=0.30)	Pass
PBT vs all towers	Baseline + 19 variables	0.121 (p=4.4 × 10^{-4})	−0.050 (p=0.15)	0.036 (p=0.30)	−0.003 (p=0.92)	Pass
GBM vs all towers	Baseline	0.135 (p=5.5 × 10^{-5})	0.075 (p=0.031)	0.058 (p=0.10)	0.088 (p=0.0088)	Fail
GBM vs all towers	Baseline + 19 variables	0.083 (p=0.016)	0.010 (p=0.79)	0.006 (p=0.86)	0.029 (p=0.40)	Pass
Non-GBM vs all towers	Baseline	0.117 (p=0.0011)	−0.028 (p=0.43)	0.051 (p=0.15)	0.006 (p=0.88)	Pass
Non-GBM vs all towers	Baseline + 19 variables	0.033 (p=0.38)	−0.103 (p=0.0048)	−0.006 (p=0.88)	−0.040 (p=0.28)	No assoc.
VS vs all towers	Baseline	0.045 (p=0.25)	0.057 (p=0.15)	−0.045 (p=0.36)	0.057 (p=0.14)	No assoc.
VS vs all towers	Baseline + 19 variables	0.024 (p=0.55)	0.045 (p=0.27)	−0.038 (p=0.44)	0.031 (p=0.44)	No assoc.
PBT vs legacy (GSM)	Baseline	0.121 (p=4.2 × 10^{-4})	−0.038 (p=0.27)	0.038 (p=0.27)	−0.030 (p=0.38)	Pass
PBT vs legacy (GSM)	Baseline + 19 variables	0.125 (p=3.4 × 10^{-4})	−0.043 (p=0.22)	0.054 (p=0.13)	−0.026 (p=0.45)	Pass
GBM vs legacy (GSM)	Baseline	0.104 (p=0.0024)	−0.023 (p=0.51)	0.066 (p=0.066)	−0.012 (p=0.72)	Pass
GBM vs legacy (GSM)	Baseline + 19 variables	0.110 (p=0.0017)	−0.022 (p=0.54)	0.061 (p=0.099)	−0.008 (p=0.81)	Pass

Association	Adjustment	2011–15 → 2016–20 (forward)	Placebo 2006–10 → 2011–15	Placebo 2001–05 → 2006–10	Reversed 2016–20 → 2011–15	Gate
VS vs legacy (GSM)	Baseline	0.012 (p=0.75)	−0.007 (p=0.87)	−0.050 (p=0.30)	0.014 (p=0.72)	No assoc.
VS vs legacy (GSM)	Baseline + 19 variables	0.016 (p=0.70)	−0.007 (p=0.86)	−0.055 (p=0.27)	0.004 (p=0.92)	No assoc.
PBT vs 2G/3G composite	Baseline	0.174 (p=1.9 × 10^{-7})	0.063 (p=0.064)	0.115 (p=7.3 × 10^{-4})	0.129 (p=1.2 × 10^{-4})	Fail
PBT vs 2G/3G composite	Baseline + 19 variables	0.094 (p=0.0062)	−0.016 (p=0.64)	0.070 (p=0.043)	0.084 (p=0.014)	Fail
GBM vs 2G/3G composite	Baseline	0.170 (p=3.8 × 10^{-7})	0.146 (p=2.5 × 10^{-5})	0.153 (p=1.7 × 10^{-5})	0.206 (p=6.7 × 10^{-10})	Fail
GBM vs 2G/3G composite	Baseline + 19 variables	0.124 (p=3.2 × 10^{-4})	0.070 (p=0.049)	0.083 (p=0.023)	0.163 (p=2.0 × 10^{-6})	Fail
PBT vs CDMA	Baseline	0.095 (p=0.0049)	0.079 (p=0.02)	0.040 (p=0.24)	0.043 (p=0.21)	Fail
PBT vs CDMA	Baseline + 19 variables	0.032 (p=0.35)	0.014 (p=0.69)	−0.005 (p=0.88)	−0.005 (p=0.90)	No assoc.
GBM vs CDMA	Baseline	0.059 (p=0.079)	0.109 (p=0.0018)	0.069 (p=0.054)	0.042 (p=0.21)	No assoc.
GBM vs CDMA	Baseline + 19 variables	0.005 (p=0.89)	0.051 (p=0.15)	0.003 (p=0.92)	−0.012 (p=0.74)	No assoc.
PBT vs UMTS	Baseline	0.091 (p=0.0073)	0.116 (p=7.1 × 10^{-4})	0.173 (p=4.1 × 10^{-7})	0.098 (p=0.004)	Fail
PBT vs UMTS	Baseline + 19 variables	−0.005 (p=0.89)	0.030 (p=0.39)	0.118 (p=7.3 × 10^{-4})	0.023 (p=0.51)	No assoc.
GBM vs UMTS	Baseline	0.114 (p=7.9 × 10^{-4})	0.135 (p=1.2 × 10^{-4})	0.205 (p=9.1 × 10^{-9})	0.139 (p=4.2 × 10^{-5})	Fail
GBM vs UMTS	Baseline + 19 variables	0.043 (p=0.21)	0.058 (p=0.11)	0.125 (p=6.1 × 10^{-4})	0.054 (p=0.12)	No assoc.

Table S2: Sensitivity analyses for PBT–all towers, GBM–all towers and GBM–legacy (GSM). The first two rows report correlations using directly age-standardized rates (19 SEER age groups, 2000 US standard population), with baseline adjustment and with baseline plus the 19 variables, respectively. Negative-binomial count models report log incidence-rate ratios per unit log density ratio, with log person-years offsets and log baseline rate adjustment, with and without the 19 covariates. Additional analyses report population-weighted baseline-adjusted first-difference slopes and partial correlations on the log scale adjusted for log baseline incidence. Estimates, 95% CIs and two-sided p-values are reported.

Analysis	PBT vs all towers	GBM vs all towers	GBM vs legacy (GSM)
Directly age-standardized rates (US 2000 standard), baseline	0.135 [0.070, 0.200], p=5.4 × 10^{-5}	0.127 [0.062, 0.192], p=1.5 × 10^{-4}	0.134 [0.067, 0.199], p=8.7 × 10^{-5}

Analysis	PBT vs all towers	GBM vs all towers	GBM vs legacy (GSM)
Directly age-standardized rates, baseline + 19 variables	0.081 [0.014, 0.148], p=0.018	0.084 [0.016, 0.150], p=0.015	0.137 [0.069, 0.203], p=9.1 × 10^{-5}
Negative-binomial count model + baseline	0.119 [0.047, 0.191], p=0.0012	0.125 [0.028, 0.223], p=0.012	−0.024 [−0.079, 0.030], p=0.38
Negative-binomial count model + baseline + 19 variables	0.055 [−0.020, 0.130], p=0.15	0.064 [−0.033, 0.161], p=0.20	−0.017 [−0.067, 0.032], p=0.49
First difference + baseline, population-weighted	0.066 [−0.019, 0.152], p=0.13	0.021 [−0.090, 0.133], p=0.71	−0.088 [−0.150, −0.025], p=0.0062
Correlation on log scale (log ratios, log baseline)	0.079 [0.013, 0.144], p=0.019	0.073 [0.007, 0.138], p=0.03	0.016 [−0.052, 0.083], p=0.65

Table S3: Variance inflation factors (VIFs) for the 19-variable model, including the exposure ratio and baseline incidence. VIFs identify collinearity concentrated among paired primary-care physician measures and urban–rural classifications; exposure-ratio VIFs were 1.2 for all towers and 1.0 for legacy (GSM), and baseline-incidence VIFs were 1.1.

Variable	GBM vs all towers	GBM vs legacy (GSM)	PBT vs all towers
Exposure (density ratio)	1.24	1.03	1.23
Baseline incidence	1.15	1.14	1.12
NCHS urban–rural	8.25	8.33	8.26
Beale rural–urban	7.31	7.36	7.29
Median income	6.23	6.11	6.22
Poverty (%)	4.13	4.01	4.14
College (%)	4.61	4.58	4.61
Uninsured (%)	1.84	1.80	1.85
Unemployed (%)	1.35	1.35	1.35
PCP/100k (CHR)	16.05	17.75	16.04
PCP/100k (AHRF)	16.40	18.31	16.36
Hospital beds/100k	1.26	1.31	1.26
Radon zone	1.56	1.54	1.56
PM2.5	1.22	1.19	1.22
Population	1.47	1.48	1.47
Population density	1.23	1.23	1.23
Hospitals with MRI/100k	1.18	1.18	1.18
Change in % aged ≥65	1.23	1.24	1.23
Neurosurgeons/100k	2.19	2.21	2.18
Diagnostic radiologists/100k	2.68	2.77	2.68
Adult smoking (%)	2.56	2.49	2.56

Table S4: Confounder-adjusted Pearson's correlations between primary malignant brain tumor, glioblastoma (GBM), and vestibular schwannoma (VS) incidence ratios and cellular infrastructure density ratios, for the GSM + CDMA + UMTS composite as well as each tower subtype. After confounder adjustment, the GBM signal localizes to the composite and GSM; CDMA, UMTS, and LTE attenuate to non-significance for GBM. The composite failed falsification checks under both adjustments for GBM and PBT (Table S1). Bonferroni correction used four individual technology comparisons (GSM, CDMA, UMTS, LTE) within each outcome, giving p<0.0125 (0.05/4), with the composite shown against the same threshold for reference. For VS, only UMTS retains significance after adjustment. Legacy cell tower infrastructure is defined as GSM (2G).

Outcome	Technology	r (confounder-adj)	95% CI	p	n	Bonferroni (p<0.0125)
**Glioblastoma**	**2G/3G composite (GSM + CDMA + UMTS)**	**0.124**	**[0.057, 0.189]**	**3.2×10^{-4}**	**865**	***
	**GSM (2G; legacy)**	**0.110**	**[0.041, 0.177]**	**1.7×10^{-3}**	**836**	**
	CDMA (2G/3G)	0.005	[−0.063, 0.072]	0.892	860	—
	UMTS (3G)	0.043	[−0.025, 0.111]	0.214	848	—
	LTE (4G)	−0.013	[−0.081, 0.054]	0.697	858	—
**Primary malignant brain tumors**	**2G/3G composite (GSM + CDMA + UMTS)**	**0.094**	**[0.027, 0.161]**	**6.2×10^{-3}**	**865**	**
	**GSM (2G; legacy)**	**0.125**	**[0.057, 0.192]**	**3.4×10^{-4}**	**836**	***
	CDMA (2G/3G)	0.032	[−0.035, 0.100]	0.349	860	—
	UMTS (3G)	−0.005	[−0.073, 0.064]	0.894	848	—
	LTE (4G)	0.055	[−0.013, 0.122]	0.112	858	—
**Vestibular schwannoma**	2G/3G composite (GSM + CDMA + UMTS)	0.062	[−0.017, 0.140]	0.123	642	—
	GSM (2G; legacy)	0.016	[−0.063, 0.094]	0.698	637	—
	CDMA (2G/3G)	0.031	[−0.048, 0.109]	0.444	639	—
	**UMTS (3G)**	**0.127**	**[0.048, 0.204]**	**1.6×10^{-3}**	**636**	**
	LTE (4G)	−0.034	[−0.113, 0.044]	0.391	642	—

Table S5: Age-stratified GBM results. Baseline-adjusted and confounder-adjusted Pearson r (with p-value) for GBM vs legacy (GSM) cell tower density and GBM vs LTE

tower density ratios, stratified by age band (under 55 years, 55-69 years, 70+ years). Under-55: baseline-adjusted r=−0.016 (p=0.73, 95% CI −0.105 to 0.074), confounder-adjusted r=−0.034 (p=0.47, 95% CI −0.125 to 0.058). Ages 55-69: baseline-adjusted r=0.028 (p=0.47, 95% CI −0.047 to 0.102), confounder-adjusted r=0.025 (p=0.53, 95% CI −0.052 to 0.101). Ages 70+: baseline-adjusted r=0.151 ($p=1.8 \times 10^{-4}$, 95% CI 0.072 to 0.227), confounder-adjusted r=0.137 ($p=9.1 \times 10^{-4}$, 95% CI 0.056 to 0.215). LTE associations are non-significant within each age band (all p>0.05).

Age band	Exposure	r (baseline-adj)	p	r (confounder-adj)	p
<55	Legacy (GSM)	−0.016	0.73	−0.034	0.47
	LTE	0.053	0.25	0.018	0.70
**55–69**	**Legacy (GSM)**	**0.028**	**0.47**	**0.025**	**0.53**
	LTE	0.051	0.18	0.017	0.66
**70+**	**Legacy (GSM)**	**0.151**	**1.8×10^{-4}**	**0.137**	**9.1×10^{-4}**
	LTE	0.072	0.073	0.034	0.41

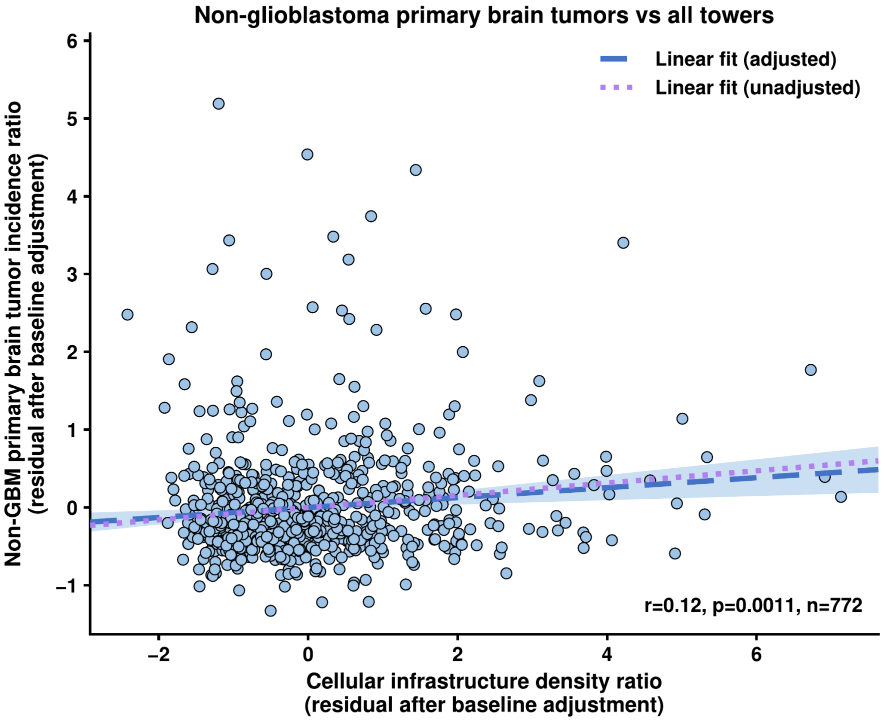

Figure S1: Primary malignant brain tumors excluding GBM. Scatter plot of incidence ratios for non-GBM primary malignant brain tumors vs all-tower infrastructure density ratios, with both axes showing residuals after baseline adjustment ($r=0.12$, $p=1.1 \times 10^{-3}$). The association attenuates to non-significance after 19-variable confounder adjustment ($r=0.033$, $p=0.38$). Blue dashed line and shaded area represent the regression fit with a 95% confidence interval; purple dotted line shows unadjusted regression.

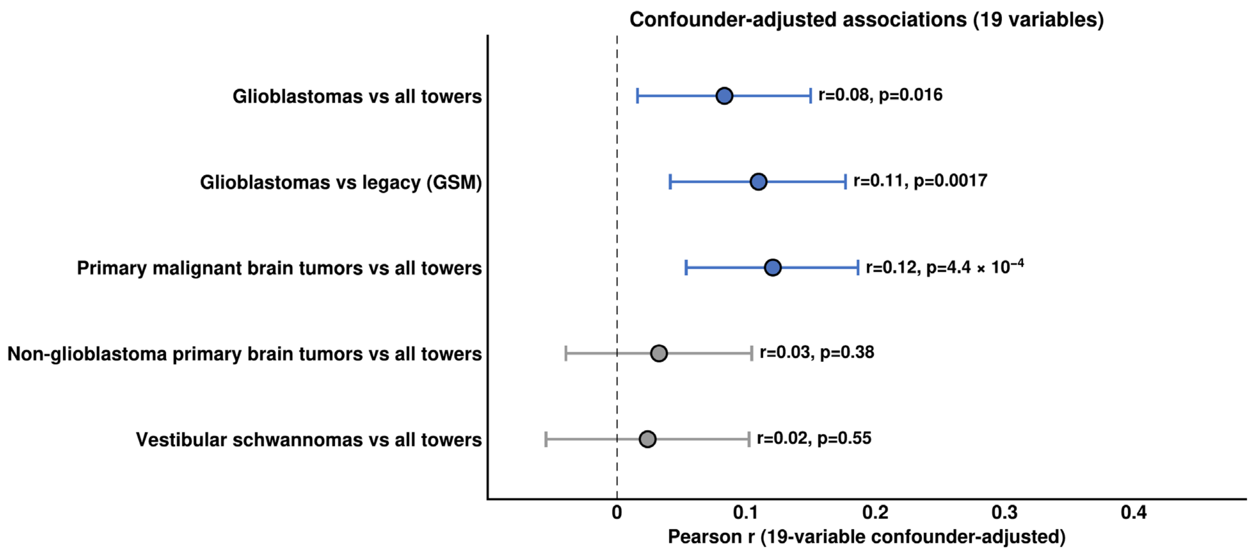

Figure S2: Confounder-adjusted associations (forest plot). Partial Pearson r with 95% confidence intervals after adjustment for all 19 county-level confounders and baseline incidence (n=865 for PBT–all and GBM–all; n=836 for GBM–GSM; n=761 for non-GBM–all; n=642 for VS–all) for: GBM vs all towers, GBM vs legacy (GSM) cell tower density, primary malignant brain tumors vs all towers, primary non-GBM tumors vs all towers, and VS vs all towers. Dashed vertical line at r=0. GBM vs all towers, GBM vs legacy

(GSM) cell tower density, and primary malignant brain tumors vs all towers remain significant; non-GBM tumors and VS are non-significant.

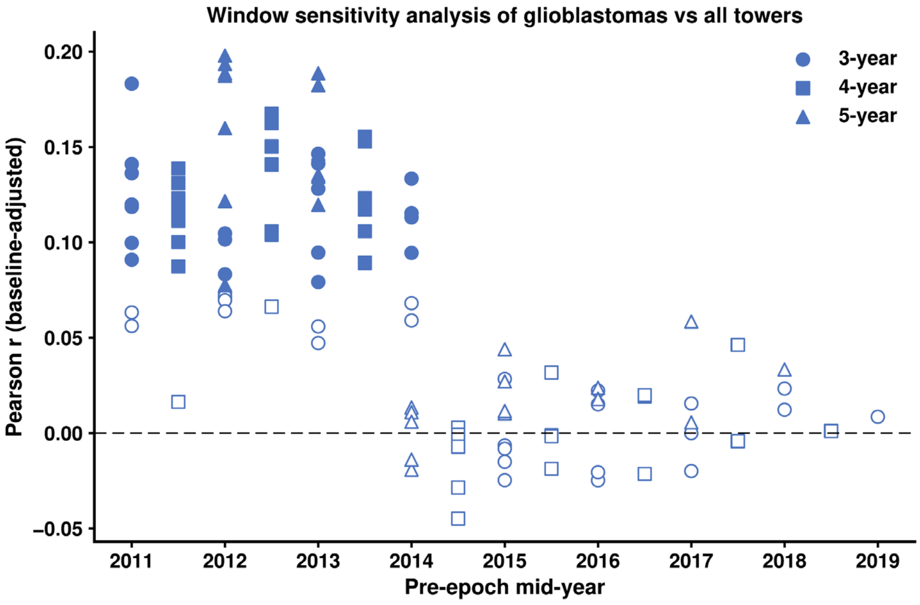

Figure S3: Deployment-era analysis (pre-epoch era varying). Baseline-adjusted Pearson r for GBM vs all-tower density across all valid epoch-window specifications, varying pre-epoch start year and window length (3-, 4-, and 5-year windows). Each specification is plotted against the mid-year of its pre-epoch. Filled symbols indicate $p<0.05$; open symbols indicate $p\geq0.05$. GSM records were too sparse before 2015 for reliable density ratios.

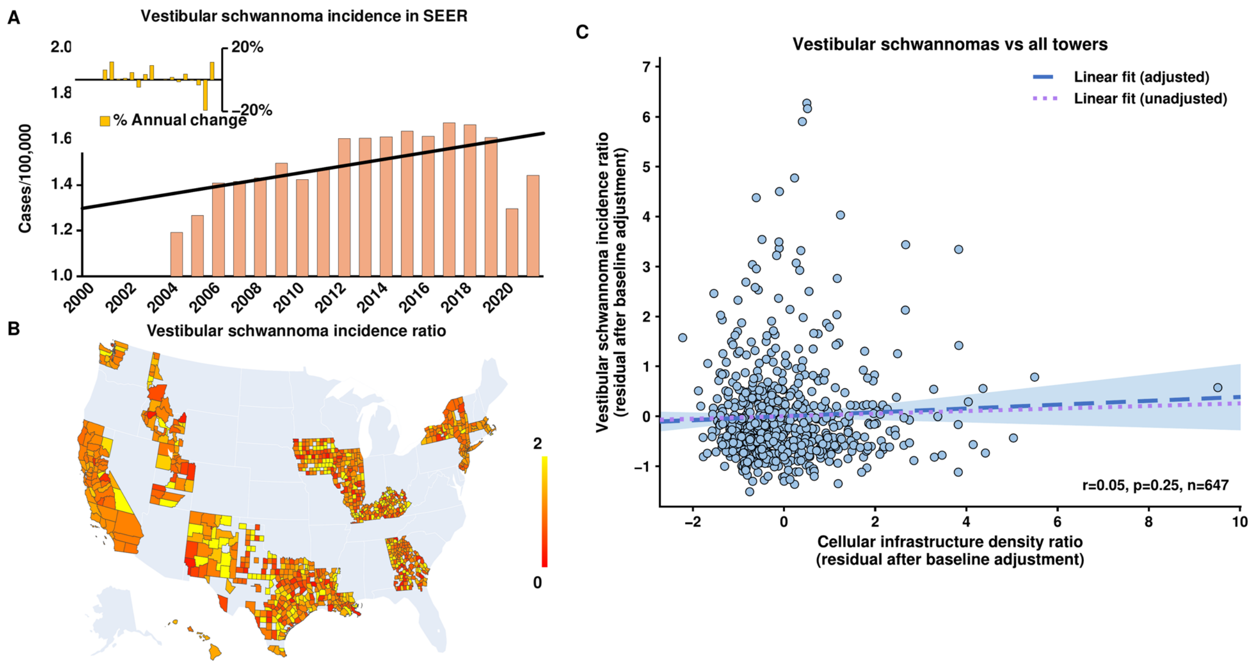

Figure S4: No significant association between cellular infrastructure growth and vestibular schwannoma incidence. (A) Annual total vestibular schwannoma (VS) incidence per 100,000 people across counties in the most recent SEER county-level data available (2000-2021) shows an annual increase of 5.6% (black trendline). **(B)** County-level variation in VS incidence growth, shown as the ratio of average cases per 100,000 people between epochs. **(C)** Scatter plot showing the relationship between county-level vestibular schwannoma (VS) incidence ratios and all-tower infrastructure density ratios, with both axes showing residuals after adjustment for baseline (2011-2015) incidence. The blue dashed line and shaded area represent a regression fit with a 95% confidence interval; the purple dotted line shows the unadjusted regression for comparison. Overall cellular infrastructure density showed no significant association with VS

incidence (baseline-adjusted $r=0.045$, $p=0.25$, $n=647$; confounder-adjusted $r=0.024$, $p=0.55$; unadjusted $r=0.028$, $p=0.48$).

Supplementary Methods

Data Sources

The Surveillance, Epidemiology, and End Results (SEER) Program of the National Cancer Institute provides population-based cancer incidence data for the United States at the county level, including detailed tumor site and morphology classifications, standardized site recode groupings, and histology/behavior codes that enable consistent analysis across cancer types. We analyzed tumor incidence data from the *SEER Research Plus Limited-Field Data, 22 Registries, Nov 2023 Sub (2000-2021)* database [19], encompassing 1104 counties and metropolitan area registries across 17 states. Using the SEER Brain and CNS Recode system, which follows the World Health Organization's International Classification of Diseases for Oncology, Third Edition (ICD-O-3) [52], we analyzed incidence data for total primary malignant brain tumor cases regardless of histological type (SEER Primary Site C70.0, C70.9-C71.9), glioblastomas (GBM; ICD-O-3 morphology codes 9440/3, 9441/3, 9442/3, and 9445/3), and vestibular schwannomas (ICD-O-3 morphology code 9560 with ICD-10-CM location C72.4-Acoustic nerve). Age-stratified incidence counts were available in SEER at 5-year age-group resolution. Incidence rates were normalized by county and year using U.S. Census Bureau intercensal county population estimates (2000-2023) [21,22]. Connecticut was excluded due to its 2019 county reorganization, Alaska's registry is not

county-coded, and Washington data cover Seattle–Puget Sound. Diagnostic methods and confirmation were not available in these data.

Cellular infrastructure location data was obtained from OpenCelliD [20], an open database of cellular towers. Each entry includes geographical coordinates, dates first added to the database, and cellular technology types, including Global System for Mobile Communications (GSM; 2G), Code-Division Multiple Access (CDMA; 2G/3G), Universal Mobile Telecommunications System (UMTS; 3G), Long Term Evolution (LTE; 4G), and New Radio (NR; 5G). OpenCelliD aggregates data from multiple sources, including crowdsourced measurements, mobile network operator submissions, and regulatory databases, with automated validation processes ensuring data quality. We mapped cell tower locations to counties using U.S. Census Bureau TIGER/Line county boundary shape files via point-in-polygon spatial analysis, then aggregated tower counts by county and year for overall cellular infrastructure as well as for each technology generation. OpenCelliD records are cell records classified by their recorded technology, with no information on operating status or upgrades.

County Covariates

PBT comprised the 15 malignant Brain and CNS Recode categories 1.1.1–1.6, with GBM corresponding to category 1.1.2. Age-specific denominators came from SEER county population estimates [53] (1990-2024). County land area came from 2023 TIGER/Line [54]. The 19 covariates, linked by county FIPS, were: 2013 NCHS urban–rural classification [55]; 2013 USDA Beale code from AHRF [56]; median household income, poverty percentage, college attainment percentage, uninsured percentage, and unemployed percentage from the 2016-2020 ACS [57]; primary care physicians per 100,000 from 2020 County Health Rankings [58]; 2020 primary care physicians excluding residents and hospital beds, 2019 short-term general hospitals with MRI, and 2015 neurosurgeons and diagnostic radiologists, each per 100,000, from AHRF [56]; adult smoking from 2016 County Health Rankings [58]; EPA radon zone [59]; mean available 2013-2017 PM2.5 highest annual average concentrations (monitor and modeled data) from CDC Tracking [60]; mean 2011-2015 population; that mean divided by land area; and post-minus-pre change in the percentage aged 65+, calculated from epoch person-years.

Study Sample

The linked county data contained 1,069 counties in 15 states after geographic exclusions, including seven counties in Georgia, Idaho, and Texas. Excluding 179 counties with zero GBM counts in either 2011-2015 or 2016-2020 left 890 counties. Multivariate outliers were identified using Mahalanobis distance computed on z-scored tower density and incidence ratios. This step excluded Miller, GA (13201), Page, IA (19145), Clearwater, ID (16035), Schuyler, NY (36097), Castro, TX (48069), and Mason, TX (48319), leaving 884 counties. County-level association analyses used this sample and required positive pre- and post-epoch outcome rates and exposure densities and finite ratios for the particular outcome and technology. All-tower PBT and GBM analyses retained 884 counties, with 865 complete on all 19 covariates after excluding 16 missing primary care physician values and three missing radon zones. GSM analyses retained 853 counties because 31 lacked GSM records in 2015, and 836 had complete covariates after a further 14 primary care physician and three radon-zone exclusions. For non-GBM PBT and VS against all towers, outcome positivity left 772 and 647 counties, respectively, and complete-case adjustment left 761 and 642. Age-band and temporal-window eligibility was reapplied for each specification, while annual panel models retained county-years with positive counts, population, and density. The sigmoid deployment fit and the annual PBT and GBM incidence curves used all 1,069 linked counties.

Exposure and Outcome Construction

The principal epochs were 2011-2015 and 2016-2020. For county i and epoch e, C_ie was the sum of diagnosis-year counts, P_ie the sum of annual population estimates, and R_ie = C_ie/P_ie the crude incidence rate, multiplied by 100,000 for presentation. Non-GBM PBT counts equaled PBT counts minus GBM counts. OpenCelliD records were grouped by state, county, year first added to the database, and recorded cellular technology, without combining co-located cells. Counts were cumulatively summed over 2005-2024. All-tower counts included every recorded technology, while individual series selected records labeled GSM, CDMA, UMTS, LTE, or NR. Density D_it equaled cumulative records in snapshot year t divided by land area in km^2, and the exposure ratio was X_i = D_i,2020/D_i,2015. The outcome ratio was Y_i = R_i,2016–2020/R_i,2011–2015. The GSM + CDMA + UMTS composite summed the three cumulative count series before dividing by area, and the post hoc legacy definition used GSM alone.

For direct age standardization, rates were calculated within 19 SEER groups comprising age 0, ages 1–4, 5–9 through 80–84, and 85+ years, then summed using normalized 2000 US standard population weights [61]. SEER population ages 85–89 and

90+ were pooled, unknown-age cases were omitted, and an age group with zero population contributed zero to the standardized rate. The standardized post/pre rate ratio and standardized baseline rate replaced their crude counterparts in the same partial-correlation procedure.

Statistical Models

Cumulative cellular infrastructure counts for 2005-2024 were fitted to $N(t) = A/[1 + \exp\{-b(t-c)\}]$ using MATLAB nonlinear least squares (nlinfit). Annual incidence was calculated as 100,000 × summed cases/summed population within each year. A straight line was fitted against calendar year, and annual percentage change was expressed as 100 × slope/mean annual incidence.

For baseline-adjusted correlations, ordinary least squares models with an intercept regressed X_i and Y_i separately on the outcome-specific pre-epoch rate, and Pearson r was calculated between the two residual vectors. With k nuisance covariates excluding the intercept, two-sided tests used $t = r\sqrt{[(n-k-2)/(1-r^2)]}$ on $n-k-2$ degrees of freedom. Fisher 95% confidence limits were $\tanh[\operatorname{atanh}(r) \pm 1.96/\sqrt{(n-k-3)}]$, with k=1 for baseline adjustment and k=20 for baseline plus the 19 covariates. Unadjusted correlations used k=0, and the secondary PBT analysis additionally residualized both

variables on the GBM incidence ratio. The 19-variable model included all covariates listed above simultaneously with baseline incidence. Missing covariates were handled by complete-case exclusion, with numeric ACS annotation values below −100,000 treated as missing. Covariates entered the headline and age-specific correlation residualizations in their original units, while first-difference and count-model covariates were standardized by subtracting the analysis-sample mean and dividing by the sample standard deviation.

First-difference models estimated $\log(Y_i) = \alpha + \beta \log(X_i) + \varepsilon_i$, with an intercept representing common change. Adjusted versions added log pre-epoch incidence, alone or with the 19 covariates, and used HC3 heteroskedasticity-robust standard errors and 95% confidence intervals from statsmodels. The population-weighted sensitivity used weighted least squares for the baseline-adjusted log first difference, with weights equal to mean annual county population over 2011-2020 and HC3 inference. The log-scale correlation sensitivity instead correlated residualized $\log(X_i)$ and $\log(Y_i)$, adjusting for log baseline incidence with the same t tests and Fisher intervals.

Negative-binomial models estimated $\log E(C_{i,post}) = \log(P_{i,post}) + \alpha + \beta \log(X_i) + \gamma \log(R_{i,pre})$, with or without the 19 standardized covariates. Post-epoch

person-years were the summed annual county population estimates used for the crude rates, and log($P_{i,post}$) was an offset with coefficient fixed at one. The statsmodels discrete NegativeBinomial fit estimated dispersion by maximum likelihood, with a maximum of 200 iterations, and returned model-based standard errors, 95% Wald confidence intervals, and two-sided p-values for β. These models used the same eligible samples as the corresponding ratio analyses.

Annual two-way fixed effects estimated $\log(C_{it}/pop_{it}) = a_i + \lambda_t + \beta \log(D_{it}) + \varepsilon_{it}$ over 2010-2021, with county effects a_i, year effects λ_t, and county-clustered standard errors and 95% confidence intervals returned by linearmodels PanelOLS. The unbalanced panels for PBT–all, GBM–all, and GBM–GSM contained 8,225, 7,021, and 5,010 county-years, respectively, after positivity filtering. GBM age-stratified correlations used bands younger than 55, 55–69, and 70+ years, with band-specific cases, person-years, baseline rates, and positivity requirements, and either baseline-only or full covariate adjustment. The same partial-correlation tests and Fisher confidence intervals were applied within each band.

Figure 4 fixed the pre-epoch at 2010-2014 and advanced the start of a 5-year post-epoch from 2011 through 2017, giving start-year separations of 1–7 years. Each

specification used densities at the two epoch endpoints and adjusted both ratios for the pre-epoch outcome rate, with specification-specific eligibility and Fisher confidence intervals. Separations of 1–4 years produced overlapping incidence windows. The deployment-window grid enumerated lengths $w=3$, 4, or 5, pre-epoch starts $a \geq 2010$, and post-epoch starts $c>a$ satisfying $c+w-1 \leq 2021$, yielding 109 specifications per pairing. The same baseline-adjusted correlation was applied to GBM and PBT against all towers, and Figure S3 displayed GBM estimates against $a+(w-1)/2$, with filled symbols for $p<0.05$. Figure 4 displayed the two all-tower pairings.

VIFs were the diagonal elements of the inverse correlation matrix of the exposure ratio, baseline rate, and all 19 covariates on each complete-case sample. Residual spatial autocorrelation used Moran's I on residuals from Y_i regressed on X_i, baseline rate, and, where applicable, the 19 covariates, with row-standardized queen contiguity from 2023 TIGER/Line county polygons, 9,999 permutations, and random seed 20261001. Scatter-plot confidence bands used the usual residual-regression mean-response interval with t critical values on $n-2$ degrees of freedom.

Falsification Checks

For Table S1, exposure remained the 2020/2015 density ratio in every run. Forward incidence change was 2016-2020/2011-2015, placebo A was 2011-2015/2006-2010, placebo B was 2006-2010/2001-2005, and time reversal was 2011-2015/2016-2020. Both variables were residualized on the denominator epoch's incidence rate, alone and with the 19 covariates, requiring positive finite outcome rates and an available exposure ratio for each run. For these checks, the 19 covariates were standardized across the 884-county sample before complete-case filtering. Population denominators used U.S. Census Bureau county population estimates for 2010-2020 and SEER county population estimates for 2001-2009. PASS required $r>0$ and two-sided $p<0.05$ in the forward run and no significantly positive result under the same criterion in placebo A, placebo B, or reversal. The rule was evaluated separately for baseline-only and full covariate adjustment.

Multiple Comparisons

Correlation and regression tests were two-sided, with $p<0.05$ used unless otherwise specified. Bonferroni correction covered four individual technologies (GSM, CDMA, UMTS, LTE) within each outcome, giving $p<0.0125$ (0.05/4), with the composite displayed against that threshold for reference.

Software and Code

The recorded Python environment was Python 3.10.9 with NumPy 2.1.3, pandas 2.2.3, SciPy 1.15.3, statsmodels 0.14.6, linearmodels 7.0, libpysal 4.13.0, and esda 2.7.0. Analysis code and derived data will be deposited in Zenodo, as stated in the Data Availability Statement.

References

19. Surveillance, Epidemiology, and End Results (SEER) Program. SEER*Stat Database: Incidence—SEER Research Plus Limited-Field Data, 22 Registries, Nov 2023 Sub (2000–2021)—Linked To County Attributes—Time Dependent (1990–2022) Income/Rurality, 1969–2022 Counties. National Cancer Institute, DCCPS, Surveillance Research Program, Released April 2024, Based on the November 2023 Submission. Available online: https://www.seer.cancer.gov (accessed on 20 August 2024).

20. OpenCelliD. Cell Tower Database. Available online: https://www.opencellid.org/ (accessed on 12 May 2024).

21. U.S. Census Bureau. County Population Totals: 2010–2019. Available online: https://www2.census.gov/programs-surveys/popest/datasets/2010-2019/counties/totals/ (accessed on 19 October 2026).

22. U.S. Census Bureau. County Population Totals and Components of Change: 2020–2023. Available online: https://www.census.gov/data/tables/time-series/demo/popest/2020s-counties-total.html (accessed on 12 June 2024).

52. Forjaz, G.; Barnholtz-Sloan, J.S.; Kruchko, C.; Siegel, R.; Negoita, S.; Ostrom, Q.T.; Dickie, L.; Ruhl, J.; Van Dyke, A.; Patil, N.; et al. An Updated Histology Recode for the Analysis of Primary Malignant and Nonmalignant Brain and Other Central Nervous System Tumors in the Surveillance, Epidemiology, and End Results Program. Neuro-Oncol. Adv. 2021, 3, vdaa175. https://doi.org/10.1093/noajnl/vdaa175.

53. Surveillance, Epidemiology, and End Results (SEER) Program. U.S. County Population Data, 1990–2024, 20 Age Groups, Adjusted. Available online: https://seer.cancer.gov/popdata/yr1990_2024.20ages/us.1990_2024.20ages.adjusted.txt.gz (accessed on 19 October 2026).

54. U.S. Census Bureau. TIGER/Line Shapefile, 2023, Nation, U.S., County and Equivalent Entities. Available online: https://www2.census.gov/geo/tiger/TIGER2023/COUNTY/tl_2023_us_county.zip (accessed on 19 October 2026).

55. National Center for Health Statistics. 2013 Urban–Rural Classification Scheme for Counties. Available online: https://www.cdc.gov/nchs/data-analysis-tools/urban-rural.html (accessed on 19 October 2026).

56. Health Resources and Services Administration. Area Health Resources Files, 2020–2021 and 2022–2023 Releases. Available online: https://data.hrsa.gov/topics/health-workforce/ahrf (accessed on 19 October 2026).

57. U.S. Census Bureau. American Community Survey 2016–2020 5-Year Estimates. Available online: https://www.census.gov/data/developers/data-sets/acs-5year.html (accessed on 19 October 2026).

58. County Health Rankings & Roadmaps. 2016 and 2020 County Data. Available online: https://www.countyhealthrankings.org/health-data/methodology-and-sources/data-documentation (accessed on 19 October 2026).

59. U.S. Environmental Protection Agency. EPA Map of Radon Zones. Available online: https://www.epa.gov/radon/epa-map-radon-zones (accessed on 19 October 2026).

60. Centers for Disease Control and Prevention. Environmental Public Health Tracking Network: PM2.5 Highest Annual Average Concentration (Monitor + Modeled Data). Available online: https://ephtracking.cdc.gov/DataExplorer/ (accessed on 19 October 2026).

61. Surveillance, Epidemiology, and End Results (SEER) Program. 2000 U.S. Standard Population, 19 Age Groups. Available online: https://seer.cancer.gov/stdpopulations/ (accessed on 19 October 2026).
